# Taking the mystery away from shared intentionality: The straightforward view and its empirical implications

**DOI:** 10.3389/fpsyg.2023.1068404

**Published:** 2023-03-30

**Authors:** Stefano Vincini

**Affiliations:** ^1^Alexander von Humboldt Foundation, Bonn, Germany; ^2^Department of Philosophy and Political Science, TU Dortmund University, Dortmund, Germany

**Keywords:** shared intentionality, social cognition, phenomenology and cognitive science, developmental psychology, emotion sharing, joint attention, infant-caregiver interaction, socially extended mind

## Abstract

Ordinary language in Western and non-Western cultures individuates shared mental states or experiences as unitary interpersonal events that belong to more than one individual. However, a default assumption in modern Western thought is that, in this regard, ordinary language is either illusory or merely metaphorical: a mental state or experience can belong to only one person. This assumption is called Cartesian eliminativism and is often taken to be foundational in psychology. It follows that any view that contradicts Cartesian eliminativism is *a priori* suspected of being “mysterious,” i.e., of not meeting scientific standards. This paper suggests that the very opposite may be the case. The straightforward view explains how individuals assemble and experience a shared mental state as a unitary whole whose components are distributed among the participants. The naturalistic advantages of such a view are brought to light by focusing on developmental science. Since it explains early shared emotions, goals, and attention merely by relying on domain-general, associative processes, the straightforward view is more parsimonious than current psychological theories. Indeed, it abandons the cumbersome postulates of (i) multi-level recursive mindreading and (ii) a special, conceptually elusive phenomenal quality. I outline the distinctive developmental predictions of the view and discuss how it accounts for the functions of shared mental states. As a reductionist, non-eliminativist approach, the straightforward view promises to be viable also for cognitive scientists who have so far worked within the Cartesian framework due to a lack of a rigorous and sufficiently developed alternative.

## 1. Introduction

Ordinary language in Western and non-Western cultures individuates shared mental states or experiences as “interpersonal events” had by more than one individual (Barrett, 2017, p. 148; Carr, 1986a; Harré, 1986; Scheler, 2008; Tollefsen, 2015). For example, we talk about “our emotion,” “our goal,” “our intention,” “our attention,” etc. By means of possessive adjectives/pronouns or the notion of ownership entailed by a verb like “to have,” ordinary language refers to the “subjective character” of mental states or experiences (Husserl, 1999; Zahavi, 2014). “Max has an emotion” means that Max is the subject of the emotion. “Alex has a thought” means that Alex is the subject of the thought. When we say things like “When we received the announcement, we experienced *a* great joy…,” “we had *the intention* [no plural suffix “s”] to do that, but…,” “*our* attention was wholly grabbed by the artist’s performance…,” etc., we designate—respectively—a unitary emotion, a unitary intention, and a unitary joint attentional state, as having more than one subject.

The modern development of Western culture has led to the default assumption that ordinary language about shared experiences or mental states is either illusory or merely metaphorical. Considering its historical origins, this assumption can be called Cartesian eliminativism. Cartesian eliminativism is the assumption that a unitary experience or mental state–i.e., an experience or mental state that is numerically “one,” or “one and the same”—can only belong to a single individual (Scheler, 1973; Schmid, 2009). Since, in this context, the notion of ownership amounts to the notion of “being the subject of,” Cartesian eliminativism can also be formulated as the assumption that a unitary experience or mental state cannot have more than one subject. Obviously, this assumption has had a great influence on Western science of the mind. In psychology, Cartesian eliminativism is often taken to be a foundational assumption (Husserl, 1962; Vincini and Staiti, 2023). This means that anything that contradicts Cartesian eliminativism cannot belong to natural science and must be “mysterious.” In other words, it is *a priori* assumed that any view that contradicts Cartesian eliminativism cannot belong to psychology and cognitive science, understood as naturalistic enterprises.

The straightforward view (Carr, 1986a,b; Eilan, 2007; Schmid, 2009) contradicts Cartesian eliminativism, as it takes a realist approach to ordinary language about shared experiences and mental states. In a nutshell, the view states—and this is why it is called “straightforward”—that shared mental states are shared in the ordinary sense of “sharing” entailed by the first meanings usually indicated in a dictionary entry, i.e., the sense in which there is one and the same entity, or process, that is owned by more than one subject. This view affirms that a shared mental state, or experience, is an overarching mental process that involves the distinct contributions of more than one individual. The central ideas of the straightforward view have been advocated by a variety of theorists, including classical and contemporary phenomenologists (Husserl, 1973a; Stein, 2000; Scheler, 2008; Walsh, 2020), contemporary analytic philosophers (Tollefsen, 2002; Campbell, 2011; Gatyas, 2022) and proponents of 4E (Embodied-Enactive-Embedded-Extended) Cognition approaches to the mind (De Jaegher and Di Paolo, 2008; Hutchins, 2014; Krueger, 2016; Theiner, 2018; Gallagher, 2020; Satne, 2021; Vincini, 2021).^1^

There are various strands of empirical research that support the straightforward view, either directly or “indirectly,” i.e., by corroborating strictly related theories on phenomena such as imitation, which are intrinsically connected with shared intentionality (Vincini and Gallagher, 2021). Direct evidence for the straightforward view comes from developmental psychology (Stern et al., 1998; Tronick et al., 1998; Rossmanith et al., 2014; Fantasia et al., 2014b), neuroscience (Schilbach et al., 2013), sociology and social psychology (Cialdini et al., 1997; Collins, 2004; Rimé, 2007; Smith et al., 2007; von Scheve and Ismer, 2013; Zickfeld et al., 2017). However, when Cartesian eliminativism is taken to be a foundational assumption, this evidence is dismissed or it is *a priori* assumed that the straightforward interpretation is incorrect.

The goal of this paper is to uncover the groundlessness of this *a priori* rejection of the straightforward view. I will seek to achieve this goal in two ways. First, I will show that the straightforward view is reductionist in the sense that matters to a naturalistic explanation of shared intentionality. Second, I will argue that the straightforward view is significantly more parsimonious than influential psychological theories of shared intentionality. This “parsimony argument” will consist in showing that the straightforward view can explain how shared mental states are individuated, i.e., assembled and experienced, by relying solely on low-level domain-general processes. Since it is widely recognized that these processes play a fundamental role in ontogeny (Lövdén et al., 2020), this paper focuses on the development of shared intentionality in infants.

The rest of the paper is divided into five sections. Section 2 discusses a representative developmental-psychological theory of shared intentionality based on Cartesian eliminativism. It explains how Cartesian eliminativism leads to two widespread theoretical-psychological postulates. Section 3 examines the neural and cognitive-psychological processes that individuate *individual* mental states and prepares the ground for a proper elucidation of the straightforward view in section 4. After having clarified the reductionist character of the straightforward view, section 4 explains how it can parsimoniously assume that the same low-level domain-general processes that individuate individual mental states individuate *shared* mental states as well. Section 5 outlines the empirical implications of the straightforward view and discusses the paper’s parsimony argument. Section 6 responds to a possible objection concerning the functions of shared mental states and thus completes the parsimony argument by underlining its significance. Overall, the goal is to advocate the naturalistic viability of the straightforward view and thus reveal the groundlessness of the Cartesian attitude that *a priori* rejects it.

## 2. Two consequences of eliminativism

Two widespread ideas in current psychological theorizing on shared intentionality are the conceptual elusiveness of “sharing” and the necessity of multi-level recursive mindreading (Jankovic and Ludwig, 2018; Rakoczy, 2018; Fiebich, 2020; Schweikard and Schmid, 2021). In order to indicate how both of these ideas ultimately derive from the Cartesian eliminativist assumption, in this section I discuss Siposova and Carpenter’s (2019) influential attempt at a systematic and philosophically informed conceptual clarification of shared intentionality. I endorse many of Siposova and Carpenter’s observations (e.g., about degrees of jointness) and distinctions (e.g., between joint attention and other kinds of social attention). Thus, one of the main reasons why I chose their contribution as a representative theory to be considered in this paper is that not only is their articulation of the consequences of Cartesian eliminativism rigorous and coherent; it also takes into account their insightful observations and distinctions.

There are also two additional reasons for examining Siposova and Carpenter’s contribution. As we shall see in Section 6, their view of shared intentionality takes Cartesian eliminativism to an admirable level of sophistication, in that it seeks to accommodate some important elements of a realist straightforward approach. Furthermore, Siposova and Carpenter identify a range of phenomena that can be targeted by a unitary account of shared intentionality: shared emotions, shared goals, shared intentions, and shared or “joint” attention. This range of phenomena is precisely the straightforward view’s scope of application in the present paper. Hence, I use the term “shared intentionality” as generally applying to this range of phenomena, in accord with the philosophical literature, as well as with some of the relevant psychological literature (e.g., Tomasello, 2016). The use of other key terms in this paper is also in line with previous literature.^2^

In considering Siposova and Carpenter’s view, I will now focus on what can be called “attention sharing” or “joint attention,” which is their primary example. Siposova and Carpenter (2019) rely on common intuitions—wisely gathered from the theoretical literature—which all boil down to the idea that a joint attentional state is not “individual,” but “shared.” In light of these intuitions, they argue that being aware of each other’s attention to the same thing—“common knowledge,” including an indefinite number of iterative levels—is not enough for sharing attention. Nor is it enough that the state of social attention is such that each individual can be in that state only if the other individual is in that state too—“ontological interdependence.” Common knowledge and ontological interdependence are *not* sufficient for joint attention, since these are essential features of another important phenomenon of social attention, which is called “common attention” and involves no sharing of attention.

In order to characterize attention sharing, Siposova and Carpenter suggest that the decisive factor for sharing is “the second person,” a notion that can be interpreted in radically different ways (Eilan, 2020). Siposova and Carpenter interpret the second person on the basis of the Cartesian eliminativist assumption that a unitary mental state can be had only by a single individual.^3^ The first consequence of this assumption can be articulated as follows.

If you interpret the second person on the basis of the assumption that a mental state can be had by only one person, it seems that, no matter how rich the communication is between you and me, and no matter how emotionally involved we are with each other, there can only be a state of attention that is only “mine,” because it can be had by only one person, and then another state of attention that is only “yours,” again because only one person can have it. In other words, on the basis of the alleged foundational assumption of psychology, it seems that, although we can add as many individual acts of communication and emotion as we like—*as far as “sharing” is concerned*—we can only have what we already have in the case of “common attention,” i.e., a phenomenon of individual attentional states that are richly interdependent on and reciprocally aware of each other. These mental states can only be *individual* states, and the sense in which they would be “shared” remains elusive.

That there is, in fact, this remaining elusiveness is confirmed by Siposova and Carpenter (2019, p. 263), who repeatedly affirm that an essential feature of attention sharing must be a distinctive phenomenal quality, where this quality is taken to be an ineffable “coloration” of experience that eludes further conceptual clarification.^4^ Importantly, the postulation of this conceptual elusiveness is representative of a large portion of current theorizing on shared intentionality: there is a widespread way of thinking—famously represented by Searle (1990)—that assumes that all we can say about the distinctiveness of the experience of sharing is that evolution has provided us with a primal and distinctive phenomenal quality that we call “sharing” or “we-ness.”

The second consequence of Cartesian eliminativism—multi-level recursive mindreading—is representative of an even larger portion of the theoretical literature; and referring here to the discussion of the mutual openness of shared experiences will suffice to indicate how it derives from Cartesian eliminativism. Like many other theorists (cf. Jankovic and Ludwig, 2018; Rakoczy, 2018; Fiebich, 2020; Schweikard and Schmid, 2021), Siposova and Carpenter assume that, in shared or joint attention, the functionally relevant features of the participants’ mental states must be “out in the open” for each participant. As Campbell (2005, 2011) explains,^5^ since Cartesian eliminativism assumes that the only mental states that participants can experience are states that are had in each case by a single individual, the only way in which the relevant features of each other’s mental states can be “out in the open” is by means of multi-level recursive mindreading. Accordingly, Siposova and Carpenter (2019, p. 264) postulate a highly complex “cumulative structure” of three levels of recursive mindreading that must occur one on top of the other.^6^ I will engage in a closer comparison between Siposova and Carpenter’s theory and the straightforward view in sections 5, 6. For now, it suffices to anticipate that the straightforward view claims that we can get rid of both recursive mindreading and the conceptually-elusive special “sharing” quality by considering how mental states are individuated, i.e., how they are assembled and experienced.

## 3. The individuation of individual mental states

The present section and the next are both dedicated to examining the individuation of experiences or mental states. Specifically, the present section is devoted to the individuation of individual mental states, whereas the next section concerns the individuation of shared mental states. Each of these sections is in turn divided into two subsections, the first subsection providing an overview of the structure of unitary mental states, and the second subsection discussing the processes underpinning individuation.

The straightforward view suggests not only that there is a structural analogy between individual and shared mental states, but also that the processes underpinning individuation are the same for individual and shared states. Therefore, it is necessary to become familiar with the structure of the individuation of individual mental states and the processes underpinning it.

### 3.1. The structure of individual mental states

The individuation of all kinds of individual mental states constitutes the background of Barrett’s (2017) constructionist theory of emotions. For this reason, it is helpful to consider the theory of such a perspicacious neuroscientist, focusing on claims of hers that could be widely accepted. Barrett’s fundamental idea is that the brain assembles (“constructs”) your experiences by integrating different components into a *pattern*. She describes the individuation of a mental state as the “categorization” of particular elements as components of a pattern:

[C]ategorization constructs *every* perception, thought, memory, and other mental event that you experience, so *of course* you construct instances of emotion in the same manner. [...] I’m speaking of the rapid, automatic categorization performed constantly by your brain, in every waking moment, in milliseconds, to predict and explain the sensory input that you encounter. Categorization is business as usual for your brain… (Barrett, 2017, p. 86)

Importantly, Barrett (2017, pp. 95, 96) explains that, through “statistical learning,” a human brain assembles experiential patterns from “a very young age,” when many theorists would not speak of “categorization.” However, the idea that the brain assembles these kinds of *basic or more complex* patterns is not controversial in contemporary neuroscience (Prochazkova and Kret, 2017). Therefore, if we understand Barrett’s argumentation as being broadly about *pattern formation and instantiation*, we can take her view of mental state individuation to be fairly representative.

As a first example, consider the case of early action experience. According to Delafield-Butt and Trevarthen (2015), the mid-gestational human fetus already experiences actions as purposive and coherent complexes comprising a succession of embodied experiential phases. For example, “reach-and-grasp” and “reach-to-touch” are acquired schemas where the reaching component is combined with a grasping or touching component. These primitive action patterns entail “one coherent project with a common goal” and “constitute the beginnings of conceptual development” (Delafield-Butt and Trevarthen, 2015, pp. 4, 5).

As a second example, consider Barrett’s (2017, p. 103) own description of a concrete emotional experience:

As children grow up, they […] come to realize that emotions are events that develop over time. An emotion has a beginning or cause that precedes it (“My mommy walked into the room”). Then there’s a middle, the goal itself that is happening now (“I am happy to see my mommy”). Then there’s an end, the consequence of meeting the goal, which happens later (“I’ll smile and my mommy will smile back and give me a hug”). This means that an instance of an emotion concept helps to make sense of longer continuous streams of sensory input, dividing them into *distinct* events.^7^

I have emphasized the term “distinct” in the previous quote because it epitomizes the convergence between certain aspects of contemporary neuroscience and classical phenomenology. Just as we can see in Barrett’s example, for classical phenomenologists, an experience is a temporally extended whole composed of distinct perspectival components (Brough, 2011; Zahavi, 2011).

A core idea in classical phenomenology is that an experience can be pre-reflectively given to you as a temporally extended event only if each phase of the experience you live through is a distinct perspective on the whole. In this way, you can, e.g., have a sense that an emotion has just arisen, that you should avoid at least the most inconvenient of its impending behavioral externalizations, that it’s finally calming down, and that it has just passed and you are now up to something else. This is one of the core ideas of Husserl’s theory of inner time-consciousness, which has been explicitly taken up by contemporary neuroscientists (e.g., Varela, 1999; Northoff, 2016) and has obvious parallels with predictive coding approaches like Barrett’s (Lloyd, 2017).

All phenomenologists agree that the pre-reflective pre-delineation of a unitary experience is not full-fledged individuation (Brough, 2011). Pre-reflectively, experiences are not “separated as neatly from one another as coaches on a train,” yet they are implicitly and loosely demarcated as “discrete units” (Zahavi, 2011, pp. 19, 22). This means that pre-reflective experience allows for multiple ways of individuating experiences at the higher level of reflective, linguistic, and scientific practices, depending on the goals and contexts of these practices (Hutchins, 2014; Vincini, 2021).

Normally, however, these higher-level practices of individuation are not arbitrary. For example, Barrett (2017) suggests that ordinary language responds to functional features of hierarchically organized mental wholes, so that an experience can be considered part of a lower-level or a higher-level whole depending on what matters in a particular context.^8^ Furthermore, it is often clear when an experiential component is called an “experience” only for the purpose of philosophical and scientific analyses. For instance, we can label as an “experience” the perception of each single word of a sentence in ordinary conversation, or the presentation of each part of the large sofa I see when I get home at night. In these cases, it is clear that these “experiences” are only components of larger wholes that are pre-reflectively pre-delineated as unities, and which play concrete functional roles in our lives (Vincini, in press).

### 3.2. The processes underpinning the individuation of individual mental states

Barrett’s constructionist theory of mental states as “events that develop over time” and the classical-phenomenological theory of the individuation of experiences are both based on the idea that what a mental state or experience is—its “ontology”—is not independent of how it is experienced and/or conceptualized. The detection of a pattern in a particular situation—e.g., the pre-delineation of the anger I am experiencing, including its impending and inconvenient externalizations—is nothing other than the activation of the pattern itself, and therefore it also constitutes a tendency to realize it. This tendency can be more or less difficult to restrain, but in any case, it must always adapt to the particular circumstance.

From this perspective, when we investigate the neural and the cognitive-psychological processes that are responsible for the individuation of mental states or experiences, we certainly seek to identify processes that can at least in principle account for the original formation of a pattern, i.e., for the unification of different components that does not rely on a pattern created on a previous occasion. However, pattern formation is a continuous process of modification, because any pattern must accommodate the particular circumstance every time it is instantiated; in this manner, it continuously modifies itself by acquiring, discounting, bypassing, etc., novel or old features (Husserl, 1999; Barrett, 2017). Therefore, the examination of the processes underpinning the individuation of experiences or mental states must contain at least an implicit reference to how patterns are instantiated, and are thus continuously accommodated and modified.

Given the distinction between pre-reflective pre-delineation and full-fledged reflective individuation, it is important to differentiate between (a) low-level processes that operate at the fundamental level of original pattern formation and, generally, in our pre-reflective engagement with the world, and (b) higher-level linguistic processes that operate at the more specific level of when we reflectively turn to our experiences or mental states themselves. As anticipated in the introduction, this paper focuses on low-level processes.^9^

At a fundamental and general level, the processes assembling experiential mental states are associative processes broadly captured by the famous Hebbian refrain that neural resources that fire together, wire together. A phenomenological-psychological equivalent of this refrain would be something like: “*experiencing together* gives rise to an experiential whole that has a tendency to repeat itself when one of its elements is presented;” where “experiencing together” would refer to a plurality of experiences, each of which presents its content in a way that is connected with the other experiences (Scheler, 2009; Vincini and Gallagher, 2021).

Despite the sophistication of the most recent hypotheses, there are many open questions concerning how the processes of association should be characterized and mathematically modeled (Heyes and Ray, 2000; Barrett, 2017; Vogel et al., 2019). A still unsettled issue is whether the processes of association are reducible to a single process or “law” (Hall, 1994). For the purposes of this paper, we can remain neutral on this and similar questions, and rely on two processes of association that are commonly accepted in cognitive science in order to illustrate a fairly broad range of phenomena. It suffices to be aware that the possible eventual reduction of one process to another would not amount to an elimination of the reduced process—a proof that it does not exist—but simply to showing that the reduced process is identical with a specific implementation of a broader or more fundamental process. The first associative process we discuss can be characterized as follows.

Association by Contiguity in Time: If two experiences (A and B) occur contiguously in time, then they *tend to* form an experiential unity.^10^

To take Delafield-Butt and Trevarthen’s example, if “reaching” and “grasping” occur in short succession, then they tend to form an experiential unity, which can also be more easily repeated at a later time. However, these sorts of experiential unities do not seem to be completely independent of the contents of one’s experiences. Delafield-Butt and Trevarthen (2015, p. 5) say that reaching and grasping form “one coherent project” and it seems that the fact that they may have the same target—“reaching X” and “grasping X” (e.g., the umbilical cord or the other hand)—is relevant to the formation of such a coherent unity. If one considers simultaneity as maximal contiguity, then this idea can be accommodated by pointing out that the occurrence of “X” in “grasping X” strengthens the “retention,” or working memory, of “reaching X.” This would be why “grasping X” is experienced as continuous—not only in a temporal sense—with “reaching X.”

Nonetheless, when experiences are associated in virtue of their contents, it is usual to talk about a different associative process, i.e., association by similarity. In psychology, association by similarity can be described as a sort of “factotum” of cognition (Catmur et al., 2009; Vincini and Jhang, 2018). Phenomenologists have often emphasized the role of association by similarity in the unification of experiences distributed over time. They have observed that successive phases of experience that have the same “intentional object” or some other quality in common tend to form an experiential unity (Stein, 2000; Vincini, 2021). For example, if I walk around the Eiffel tower as a solitary tourist, the experiences I have of the different sides of the tower—the different perspectives on the same intentional object—are experienced as part of a unitary perceptual activity (Carr, 1986a). However, since a characteristic feature of association by similarity seems to be that it can unify similar simultaneous experiences from a background of other simultaneous experiences, I now discuss this process in a manner that applies to both successive and simultaneous experiences.

Association by Similarity: If two (embodied) experiences (A and B) overlap in significant ways—i.e., if they share features in common that are relevant to the life of the organism—then they tend to form an experiential unity. In terms of the neural substrates of the presentation of the experiences: neural process A and neural process B tend to form a unitary neural process AB if they concur in activating a common neural resource (overlap). The activation of a common neural resource implies that process A tends to facilitate B because it activates a resource that is an integral interconnected factor within B, and likewise from B to A.^11^

**Figure 1:**
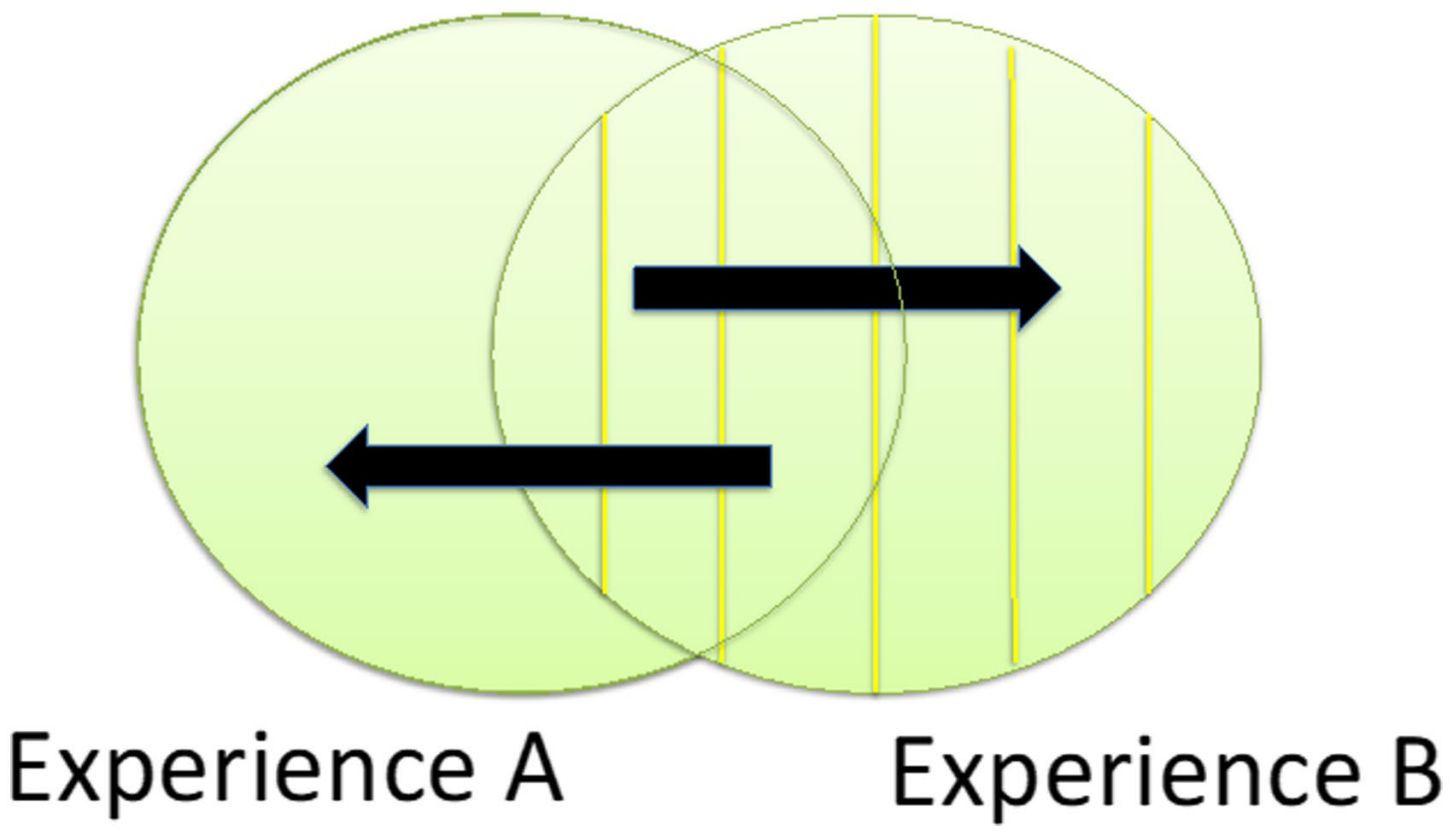
Association by similarity as process underpinning the individuation of experiences [adapted from Figure 3 in Vincini (2020)]. The two circles represent two experiences, or components of the flow of experience, and the overlap between the two circles represents what the two experiences have in common. In reference to the example of seeing two red stains on the wall, the picture highlights that while the stains on the wall do not overlap, the experiences presenting them do. At the level of the neural processes underpinning the distinct experiences, it is often legitimate to hypothesize that there is a quite literal kind of overlap (Gonzalez-Castillo et al., 2012; Barrett, 2017). As Barrett (2017, pp. 19–23) puts it, “a single brain area or network contributes to many different mental states. […] [T]he same neurons can participate in creating different mental states.” Then, the arrows moving from the overlap area to the non-overlap areas symbolize the process of reciprocal facilitation between the overlapping neural processes. In the examples from Stein (2000) and Carr (1986a) mentioned above, the individuation of an experience is connected with the individuation of the intentional object of the experience. Nevertheless, the functioning of associative links in the individuation of both the intentional object and the experience should not obscure the idea that the individuation of an intentional object and the individuation of an experience are structurally different kinds of phenomena—as emphasized by Vincini (2021).

Consider the experience of seeing two red stains on a white wall. It is not only true that the intentional object of the experience is a unitary configuration—a *Gestalt*—but also that the experience of one stain and the experience of the other stain pre-reflectively form a unity. It is only for the purpose of analysis that one distinguishes them as different experiences. Other things being equal, the similarity between experiencing a certain portion of the wall and experiencing another portion of the wall—both experiences present a red stain—tends to facilitate their integration into an experiential unity. They may form a unitary attentional process with respect to the background awareness of the wall. Indeed, the expression “seeing a pair of stains” captures the pragmatic individuation of the experience more appropriately than “seeing the wall,” although the latter is also true in this situation. “Seeing the stains” may easily lead me to ask, “Who made them? My little child?”, whereas “seeing the wall” doesn’t usually prompt a question of this kind. The same process of pre-reflective unification applies to experiences of different portions of a single stain, or to my simultaneous experiences of the different parts of my large sofa when I enter my apartment at night (Figure 1).

A general consideration of emotional experiences will help to recap my foregoing examination of association by temporal contiguity and association by similarity. In cognitive science, many theorists agree that emotions are complexes of distinct components distributed across space and time: physiological responses, action tendencies, bodily expressions, and cognitive and attitudinal components (Newen et al., 2015; Gallese and Caruana, 2016; Barrett, 2017). The experiences and the neural substrates of these components (i) are contiguous in time, and (ii) can overlap with each other, both in the case in which they succeed one another and in the case in which they are simultaneous. In this way, they “wire together,” i.e., they form patterns that regulate the pre-reflective pre-delineation of unitary experiences in subsequent instantiations. Every instantiation is always unique because it is the result of accommodation to a particular circumstance (Husserl, 1999). In Barrett’s (2017) terminology, your brain “constructs” a unique and unitary emotional episode in virtue of patterns acquired in past experience. At a fundamental and general level, it is legitimate to assume that the acquisition of these patterns occurs through domain-general associative processes like association by contiguity and similarity.

## 4. The individuation of shared mental states

### 4.1. The structure of shared mental states

Moving from individual to shared mental states, the straightforward view affirms that all kinds of mental states are unities of distinct components. Just as individual mental states are unities of individual components that are distributed across space and time, so shared mental states are unities of individual components distributed across space and time. The only difference is that, in the former case, the components all pertain to a single individual, whereas in the latter case, the components pertain to different individuals.

On the straightforward view, mental states are shared precisely in the sense of “sharing” entailed by the first meanings usually indicated in a dictionary entry, i.e., the sense in which there is *one and the same entity that stands in a relation of ownership with more than one subject.* As Salice (2015) pointed out, all sorts of entities can be shared in this ordinary sense. For example, two children can share a toy, but two people can also share a right or a debt. This ontological diversity hints at the idea that the straightforward view entails no reification of experience. Just as ownership does not reify experience in the individual case (Brough, 2011; Zahavi, 2011), nor does co-ownership do so in the shared case (Husserl, 1973a; Carr, 1986a,b; Scheler, 2008; Schmid, 2009). Indeed, most if not all advocates of the straightforward view (e.g., Hutchins, 2014; Krueger, 2016; Gallagher, 2020; Vincini, 2021) claim that, strictly speaking, “mental state” is a misnomer and that mental “states” are actually “processes.”

**Figure 2:**
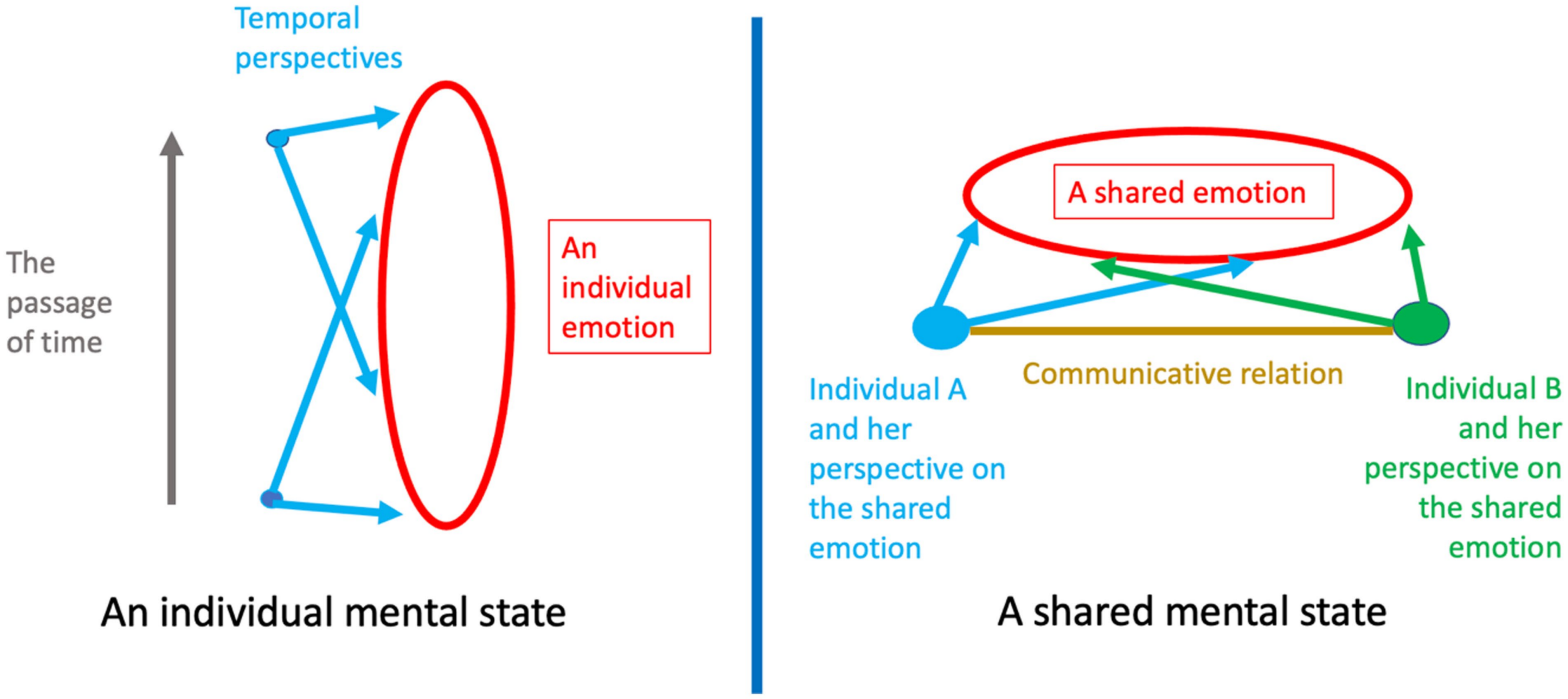
The structural analogy between individual and shared mental states. The elongated circle on the left represents an individual mental state. I take here an individual emotion as an example. The vertical axis along which the elongated figure unfolds represents the passage of time, signaling that the emotional episode is a process that lasts over time. At each point of time, the individual has a perspective on the whole of the emotion. Each of these perspectives is *eo ipso* a component, or factor, of the emotional process. For the sake of clarity, the graph shows only the perspectives that the individual has at an initial and a final point of the emotional episode—two couples of bifurcating arrows, the scope of which includes the entire emotional event, albeit from a specific angle. The most voluminous of the curved figures on the right represents a shared mental state. Again, I use a shared emotion as an example. The curved figure unfolds along the horizontal line, but it is implicitly assumed that a shared state is a process enduring over time just like an individual state. The minor circles represent two distinct individuals participating in the shared emotion (individual A and individual B). Each individual has a perspective on the emotional episode—a couple of arrows the scope of which includes the entire emotion, albeit, for each individual, from a specific angle. Each individual perspective is *eo ipso* a component, or factor, of the unitary process. The line connecting individual A and individual B represents the communicative relation subsisting between the two. An exchange of mutual looks or other reciprocal “communicative bids” can bring about the shared state (as explained in sections 4.2, 6). The structural analogy between individual and shared mental states is not restricted to emotions. For examples of perceptual, attentional states emphasizing their endurance over time in the individual case and their distribution across different individual perspectives in the shared case see Stein (2000), Carr (1986a), and Campbell (2002, 2005). For the structural analogy between individual and shared goals/intentions see Bratman (1993), Pacherie (2013), Sinigaglia and Butterfill (2022), and the examples of individual and joint actions provided in section 6.

Terminological differences aside,^12^ the straightforward view elucidates shared mental states in light of the structure that is already known in the case of individual mental states. Just as individual states must be constituted by distinct perspectival components—otherwise they could not be experienced as temporally extended events—so shared mental states must be constituted by the distinct perspectives of the participants. Otherwise, they could not be experienced as “shared” (Figure 2).^13^

Furthermore, also in the case of shared mental states, the distinct elements must have context-dependent significant features in common in order for them to be grouped as components of one and the same mental state. Such an interplay of *distinctness and similarity* can also be observed at the level of the neural underpinnings of a joint action. When performing a joint action with another person, the brain partly reuses the same resources employed in one’s own actions in order to substantiate the understanding of the components of the joint action that are carried out by the other person. The reuse of neural resources for actions executed by the self to understand actions executed by others—the self-other similarity or “overlap”—is only partial, because the brain must label the actions of others as belonging to others. Otherwise, the required coordination would be impossible (Rizzolatti and Sinigaglia, 2016; Barrett, 2017; Sebanz and Knoblich, 2021; Sinigaglia and Butterfill, 2022).^14^

The straightforward view is a form of non-eliminativist reductionism. It is non-eliminativist because, contrary to Cartesian eliminativism, but in accord with ordinary language, it implies that there are unitary mental states or experiences that are had by more than one individual (see the Introduction). However, the straightforward view is reductionist in the sense that matters for a naturalist explanation of shared intentionality: it states that shared mental states are nothing other than individual components that cognitively relate to and causally interact with each other. Since individual components, cognitive relations, and causal interactions are the ordinary elements of a naturalistic explanation, a view that reduces shared intentionality to nothing other than these elements is surely a naturalistic view. As Schmid (2009, p. 81) puts it in relation to emotion sharing, “the shared feeling is nothing in addition to what the participating individuals feel” and, more generally, shared intentionality is nothing other than a phenomenon of “interrelated individuals” or “minds-in-relations” (Schmid, 2009, p. 156).^15^^,^ ^16^

### 4.2. The processes underpinning the individuation of shared mental states

The straightforward view suggests that, at a fundamental and general level, the cognitive-psychological and neural processes that underpin the assembling and experiencing of shared mental states are the same associative processes that underpin the individuation of individual mental states. Therefore, the goal of this subsection is to clarify how the cognitive-psychological and neural processes described in section 3.2 can individuate shared mental states. Notably, the straightforward view also suggests that the inputs of these processes are of the same kind in the individual and the shared case. Thus, it is helpful to start this section with a discussion of the kind of input that is processed in the individual case. This will help us to understand how this kind of input is assembled into a unitary pattern in the shared case.

The inputs that enter a process of configuring a unitary mental pattern in the individual case belong to the kind “embodied experiences,” or “embodied mental states,” in the broad sense of experiences, or states, that present themselves as pertaining to a particular bodily subject. For example, in section 3.2 we mentioned how reaching and grasping prenatally become a unitary project by occurring contiguously in time or by having the same intentional target (Delafield-Butt and Trevarthen, 2015). Now, reaching and grasping are proprioceptively experienced, which means that they are experienced as pertaining to one’s own lived body—the “proprio” of proprioception. The organism senses them “from within.” Developmental studies suggest that, through goal-directed movements and perceptions, human fetuses and infants develop an early sense of their lived body and can differentiate what pertains to it from environmental stimuli, including social stimuli: a sense of an interoceptive-proprioceptive space distinct from the space of audition or vision (Rochat, 2003; Fagard et al., 2018; Corbetta, 2021).

Phenomenologists have extensively argued that the lived body corresponds to a minimal embodied sense of self, and that this sense of self is ultimately an intrinsic aspect of all the individual experiences that one can go through (Zahavi, 2014; Gallagher and Zahavi, 2020). For our purposes, the cognitive-scientific equivalent of the phenomenological description of the minimal embodied self is the plausible assumption that the organism is “able to distinguish, across a fairly broad range, sensory inputs resulting from the physical state and operations of its own body, from sensory input originating elsewhere” (Heyes, 1994, p. 915). This self-world differentiation applies to a large variety of organisms because it seems to be necessary for basic adaptive behaviors such as avoiding collisions or obtaining the encounter with entities in the environment. Consequently, it is legitimate to assume that an infant can differentiate between the embodied experiences of the self and embodied experiences pertaining to individuals other than the self. When an infant experiences her own reaching and grasping, she experiences them “from within,” as pertaining to the egocentric “here” of her own lived body; when she sees the reaching and grasping of the caregiver, she experiences these as “from outside:” as occurring or originating from “over there.”

For an experience or mental state, presenting itself as belonging to a particular bodily subject means having a particular “mode of presentation.” The inputs of the process of individuation of individual mental states have a particular mode of presentation because they are experiences or states that present themselves as pertaining to a particular bodily subject: the self. Now, the inputs that enter the process of configuring a shared mental state are either experiences or states presenting themselves as belonging to the self or experiences or states presenting themselves as belonging to a particular bodily subject distinct from the self. In any case, the input of the process that individuates a shared mental state is of the same kind as the input of the process that individuates an individual mental state: it is always an experience or state that presents itself as belonging to a particular bodily subject, since both self and others are particular bodily subjects. The input of a process that configures a shared mental state is an experience or state with a particular mode of presentation, just like the input of the process that configures a unitary individual mental state.

Obviously, the fact that the inputs are of the same kind in the individual and the shared case does not entail that there are no differences between the two cases. In the case of a process that configures an individual mental pattern, the input comprises only experiences and states that present themselves as pertaining to the self. In contrast, in a process that configures a shared mental pattern, the input comprises both experiences and states that present themselves as pertaining to the self, and experiences and states that present themselves as pertaining to others. We can say that the case of the individual pattern accepts input of only a specific subspecies, whereas the case of the shared pattern requires input from different subspecies.

Importantly, in the case of a shared experience, the fact that the input must comprise both experiences presenting themselves as belonging to the self and experiences presenting themselves as belonging to the other is not an obstacle to the formation of a unitary pattern. On the contrary, just as the input needed to configurate individual mental events must comprise experiences of the self that present themselves as occurring at different time points—otherwise these events would not be configured as temporally extended—so that fact is precisely what enables the formation of a pattern that individuates an overarching experience as *shared* among more than one individual (Vincini, 2021).^17^

Neither the fact that the inputs in the individual and the shared case are of the same kind nor the fact that they are different should come as a surprise. In both the individual and the shared case, the output of the cognitive-psychological and neural processes of individuation must be of the same kind, i.e., it must be a unitary mental state or experience. However, in the individual case, the output must belong to a specific subspecies—it must be an individual mental state or experience—whereas, in the shared case, the output must belong to a different subspecies—it must be a shared mental state or experience. After having identified what kinds of inputs and outputs the processes of individuation entertain, we can now examine how these processes work in the pre-reflective pre-delineation of shared mental states.

It is helpful to start with the process of association by similarity, since it is largely uncontroversial that experiencing similar embodied experiences or states in self and other contributes to bringing about shared mental states (Salmela, 2012; Zahavi, 2019; Salice and Miyazono, 2020; Crone, 2021). As a first example, consider early emotion sharing (Tomasello, 2019). In early emotion sharing, the behaviors of self and other have some characteristic features in common: the bodily actions and vocalizations of self and other are similar, they play comparable causal roles (e.g., initiating or varying the tone of the interaction), they are both regulated by a “turn-taking” structure, they both have a “response” character,^18^ they are experienced as having the same goal (e.g., prolonging or reinitiating the interaction),^19^ etc. These self-other similarities are usually called “affect attunement,” an expression that emphasizes the *intermodal* character of many features that self and other have in common (Stern, 1990; Stern et al., 1998).

The similar behaviors of self and others are expressive of the mental life of self and others. Indeed, they are what we have called “embodied experiences.” As both philosophers and developmental psychologists have argued (Stern et al., 1998; Tronick et al., 1998; Eilan, 2007; Fuchs and De Jaegher, 2009; Hobson and Hobson, 2011; Krueger, 2016), when infant and caregiver participate in emotion sharing, none of them experiences two numerically distinct emotional events; rather, each of them experiences a global emotional event, which includes both what she experiences in the egocentric “here” of her own lived body and what the other participant in the “there” of visual space experiences from his perspective.

This is easy to see in the case of the infant, who, in the excitement of a playful interaction, surely does not have the reflective-analytical capacity to identify two numerically distinct emotions—her own excitement and the excitement of the caregiver. Naturally, the straightforward view would grant that, in the case of a caregiver raised in a “WEIRD” (Western, Educated, Industrialized, Rich, and Democratic) culture and accustomed to think about the mind on the basis of the assumption that a unitary mental state can be had by only a single individual, this caregiver could reflectively individuate two numerically distinct “excitement*s*.” However, supporters of the straightforward view (Husserl, 1973a; Carr, 1986a,b; Stein, 2000; etc.) would point out that (i) the reflective thought of this caregiver would not be in line with his pre-reflective experience, (ii) it would not be in line with his ordinary language, and (iii) it would not capture the concrete function that shared emotions play in his social life as in the life of most humans—the real phenomenon that cognitive science is after (see section 6).

Why, according to the straightforward view, do infant and caregiver pre-reflectively experience the embodied experiences of self and other as spatially distinct constituents of a global emotional event? The first reason for this can be found in the unhindered functioning of association by similarity. In describing this associative process, section 3.2 stated that “if two (embodied) experiences (A and B) overlap in significant ways […] they tend to form an experiential unity.” The embodied experiences of infant and caregiver overlap in the multiple ways mentioned above. Therefore, they naturally tend to be experienced as constituents of a social and embodied event, e.g., the excitement *we* are experiencing (Stein, 2000).

**Figure 3:**
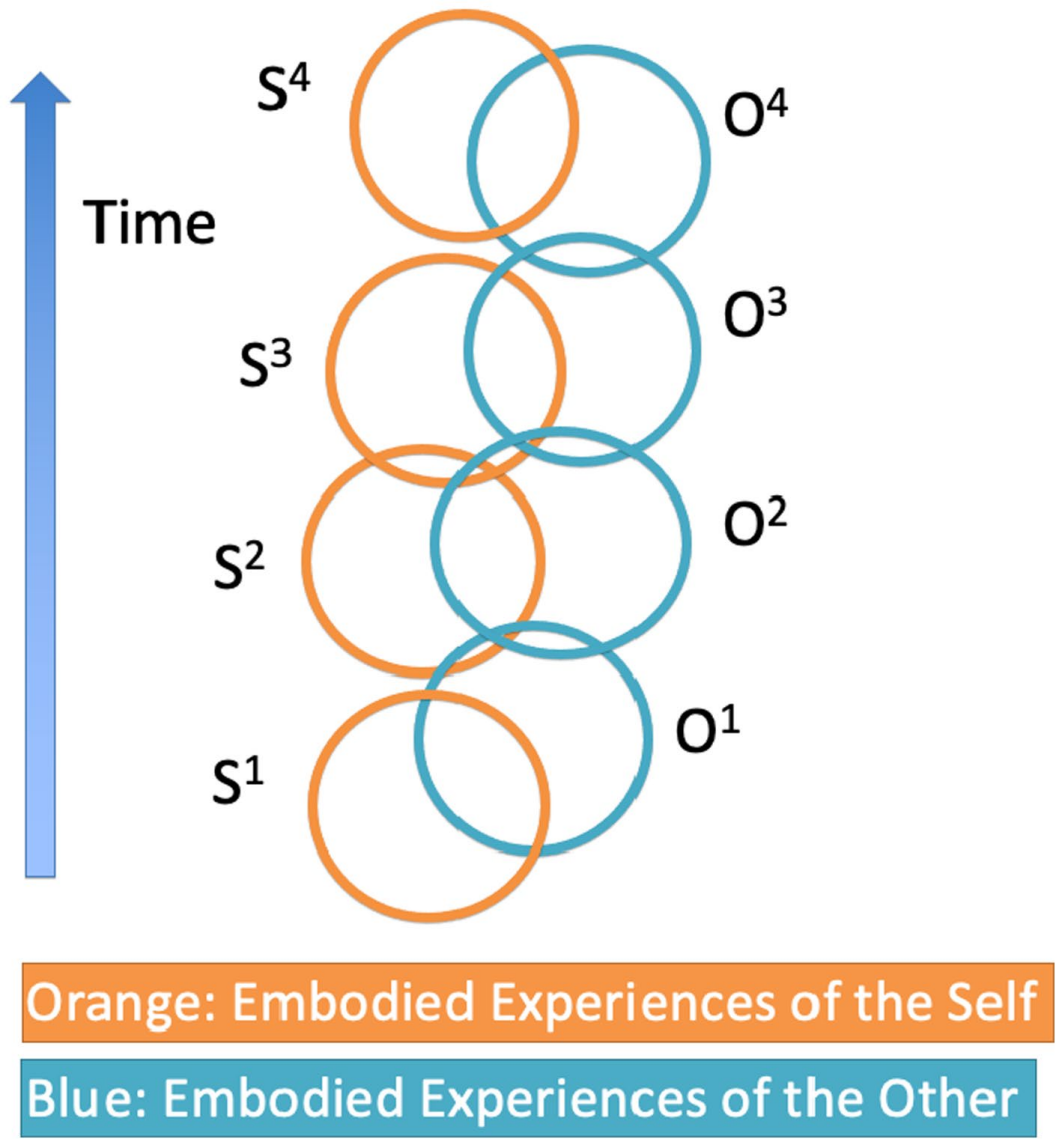
Association by similarity in the formation of shared mental states [adapted from Figure 2 in Vincini et al. (2017)]. The orange circles represent the experiences of an individual that present themselves as pertaining to her embodied self. The blue circles represent the experiences that present themselves to the same individual, but this time as belonging to a different embodied subject. Each experience of the self has a significant overlap with a corresponding experience of the other. The features that the experiences have in common can be morpho-kinetic features of the expressive behaviors of self and other, vocalizations, having the same goal, and other kinds of features that should be investigated in detail beyond the usual assumption that similarities play a role in the formation of shared intentionality. Because of their similarity or overlap, the experiences of self and other tend to be experienced as part of a unitary experiential whole. Furthermore, since in social interaction the experiences of self and other have temporal links (e.g., contiguity), they configure shared states or experiences as unitary temporally extended events (e.g., S^1^-O^1^-S^2^-O^2^-S^3^-O^3^, or S^1^-O^1^-S^2^-O^2^-S^3^-O^3^-S^4^-O^4^).

In section 3.2, we also characterized association by similarity “[i]n terms of the neural substrates of the presentation of the experiences: neural process A and neural process B tend to form a unitary neural process AB if they concur in activating a common neural resource,” where “process A tends to facilitate B because it activates a resource that is an integral interconnected factor within B, and likewise from B to A.” Since contemporary neuroscience abundantly justifies the hypothesis that the neural processes underlying the presentation of the experiences of self and other overlap (Gonzalez-Castillo et al., 2012; Rizzolatti and Sinigaglia, 2016; Barrett, 2017), it is legitimate to assume that they form a unitary process in which each neural process facilitates the occurrence of the other, most typically in the form of facilitating an expectation of a response of the self or one’s partner. The functioning of association by similarity in the pre-delineation of shared mental states is depicted in Figure 3.

We can now move on to examining association by temporal contiguity. Early proto-conversations are characterized by turn-taking—hence by “proto-roles” in the interaction—regular rhythms and patterns, and the typical “four-part structure of […] vitality” as entailing “(i) ‘introduction,’ (ii) ‘development,’ (iii) ‘climax,’ and (iv) ‘resolution,’” which applies to individual experiences too (Delafield-Butt and Trevarthen, 2015, p. 4). Therefore, infants soon learn a sequence of the kind: I do S^1^, then caregiver responds with O^1^, then I respond with S^2^, then he does O^2^, and so forth (until “resolution”). Accordingly, the embodied experiences of self and other present themselves with nexuses of contiguity that the individual learns just like she learns the nexuses of solely individual experiences.

When a subject learns an individual pattern or habit, she learns that S^1^ is followed by S^2^. This means that, if S^1^ occurs, then she expects S^2^ to occur too. For example, S^1^ may be an action and S^2^ the perception of its usual effect: if S^2^ does not occur as a consequence of S^1^, then the individual may be frustrated. Now, if O^2^ occurs contiguously to S^1^, then the individual learns the nexus: later, when S^1^ takes place (in the egocentric “here” of one’s own lived body”), she expects O^2^ to take place (in the egocentric “over there” occupied by the other), and if O^2^ fails to occur, then she may be frustrated. Thus, at the phenomenological-psychological level, when the experiences that present themselves as pertaining to self and other occur contiguously, they tend to form a unitary pattern—other things being equal. Analogously, when the neural substrates of the presentations of these experiences occur contiguously, they wire together at least in the sense that a later activation of the first will tend to facilitate the “prediction” of the second, as predictive coding neuroscience usually puts it.

All this is not new in the literature on shared intentionality. For example, it is argued that, in the case of joint actions and shared goals, individuals acquire automaticities that promote reciprocal predictability and cooperation (Martens, 2021). Naturally, a shared goal or shared emotion originates from associations occurring in the minds and brains of more than one individual, because sharing requires more than one individual. For this reason, Sinigaglia and Butterfill (2022) argue that what enables a joint action and constitutes a shared goal is an “interagential structure” of motor processes, where “interagential” means that it is distributed across different individuals (see Butterfill and Sinigaglia, 2022 for further discussion of the functional considerations underlying their argument).

In relation to how shared mental states come about, it is opportune to specify the role played by mutual looks and other reciprocal communicative gestures. According to the conception of communication as “social act” (Husserl, 1973b; Schmid, 2005; Eilan, n.d., 2020; Vincini and Staiti, 2023; *cf.* Cornejo, 2008), an individual look at another person, or another individual gesture that aims to establish a connection with another person, is not an instance of communication, but rather a “communicative bid” (Vasil et al., 2020) that can engender it. As a kind of “social act,” communication occurs only when the gesture is reciprocated through some form of uptake (Schmid, 2005; Eilan, n.d., 2020). On this conception, communication itself is thought to be a joint action—a form of shared intentionality^20^—and, the fundamental nature of communication is revealed by the etymology of the word: communication is fundamentally a “community creating” act (Husserl, 1973b, p. 473), an act of “communing” (Eilan, n.d.). Communing is realizing a “thought, or experience,” that is possible only if both “you” and “I,” i.e., “we,” participate in it (Eilan, n.d., p. 15). As Eilan (n.d., p. 13) specifies, the participants in a communing act share “the *same* experience.”

How do communicative bids bring about “communing,” i.e., how do they bring about shared mental states? A communicative bid—a look, a smile, a vocalization, a pointing gesture, etc.—engenders an attuned embodied response in the addressee—a look or a smile back, an attuned vocalization, a look in the direction of the pointing, etc. This exchange is the beginning of a pattern that both interactants, e.g., infant and caregiver, have acquired.^21^ The similarity of the embodied responses and their occurrence in accord with a relatively stable pre-delineated pattern is such that interactants experience the embodied experiences of self and other as parts of a unitary mental event that develops over time: a dyadic emotion, the shared goal of playing together, the joint attentional state to a novel toy, etc. When the unfolding of the attuned pattern is interrupted, the interactants’ expectations are disappointed. For example, in a joint attention routine, 12-month-olds become disgruntled both when (i) their adult partner does not shift the focus of attention back and forth between the infant and the infant’s referent, and when (ii) the adult does not provide a symmetrical (aligned) emotional response (Liszkowski et al., 2004; Carpenter and Liebal, 2011). The idea that humans have a fundamental motivation to engage in these kinds of shared patterns has been emphasized not just by Tomasello (2019), but also in Tronick et al.’s (1998) developmental-scientific version of the straightforward view.

## 5. The empirical implications of the straightforward view

As extensively shown by Fuchs and De Jaegher (2009), Krueger (2016), and Vincini (in press), Tronick et al.’s (1998) “Dyadic Consciousness Hypothesis” is a consistent developmental-scientific articulation of the straightforward view. It posits that in early social interaction, the infant experiences a global emotional-volitive event in which both infant and caregiver take part: “a dyadic state of consciousness.” Given the functional importance and motivational centrality of such dyadic events, the direct prediction of this hypothesis is that when the unfolding of the global event is artificially interrupted, the infant is dramatically distressed. This prediction has generated the vast empirical literature on the still-face effect: from about 2–3 months of age, infants react by frowning, gazing away, losing postural control, etc. when an adult abruptly stops interacting with them (Mesman et al., 2009; Li et al., 2019). This empirical literature can be considered a substantial corroboration of the straightforward view.

Importantly, Tronick et al. (1998), Stern (1990), Papousek (2007), and Mesman et al. (2009) defend a learning account of the still-face effect where this effect is due more to breaking expectations built up through previous experience of social interaction than to replacing likable stimuli with a distressing fixity. The learning account of the still-face effect coincides with the suggestion made by the straightforward view that there is an early and fast acquisition of shared patterns through domain-general processes that associate the embodied experiences of the self with the attuned experiences of the other. The learning account is supported by the dependence of the still-face effect on infants’ previous experience with social interaction: infant responses to the still-face depend on “maternal sensitivity, infant attachment, and a variety of other infant social and nonsocial behaviors” (Mesman et al., 2009, p. 250) as well as on factors like the familiarity of the interaction partner and cultural context (Li et al., 2019).

A telling variation of this experimental paradigm has been carried out by Fantasia et al. (2014b). These researchers tested the multimodal character of 3-month-olds’ expectations on “structured game routines,” which have central developmental functions since early infancy (Fantasia et al., 2014b, p. 1). Such expectations constitute what phenomenologists would call the “pre-reflective pre-delineation” of a shared goal, including all kinds of embodied responses that having a shared goal entails. When infant and caregiver experience the usual structure of embodied responses, they experience something that we could verbally express as “we want to play our usual game and have fun together.” However, in a condition in which the caregiver’s usual responses were presented, but without sound, and another condition in which they were presented without the usual visible gestures, 3-month-olds “significantly decreased their movements, gazed away from the mother more often and decreased their positive affect display. Furthermore, they presented increased Stunned Expressions” (Fantasia et al., 2014b, p. 7).

In this experimental manipulation of game routines, “the mother had not withdrawn from the interaction and was still offering some level of stimulation.” The preservation of contingency and attunement of the maternal responses in one modality suggests that “infants were not so much affected by a lack of maternal contingency or affective attunement […] but rather by alterations of an established game structure” (Fantasia et al., 2014b, p. 7). Experiencing a unitary structure of animate responses in self and other is precisely what, according to the straightforward view, the experience of a shared goal consists in. Hence, it fully accords with the straightforward view to state that the experience of realizing a shared goal in a cooperative game allows infants to become…

capable partners in joint actions (as they recognize and have expectations on it) even without possessing higher-level social knowledge. […] The pleasure of participating seems at least partially conditional to recognizing the moves in the sequence and being therefore able to cooperate to and in it. (Fantasia et al., 2014b, p. 7)

Due to its reliance on low-level processes, the straightforward view predicts an earlier emergence of shared intentionality than what is assumed by standard developmental theories. Inspired by core ideas of the straightforward approach—as specified by authors such as De Jaegher and Di Paolo (2008)—Rossmanith et al. (2014) have verified this prediction by showing that episodes of the sharing of attention, affect, and action occur from 3-months of age in infant-caregiver book reading routines. To capture the experience that infants and their caregivers have of shared unitary wholes comprising more elementary actions, Rossmanith et al. employ the general notion of “action arc:”

The basic arc structure with a beginning, build up, climax, and resolution is ubiquitous in physiological processes, e.g., breathing, and is fundamental to action, with different actions following different dynamic trajectories. (Rossmanith et al., 2014, p. 19)

This shaping of action arcs is found across all kinds of actions and at different levels and multiple timescales within an activity, nested into one another. At a high level, the activity of book sharing as a whole can be considered as an “overarching” action arc structure defined by the physical arrangements of the pages to be turned from cover to cover. (Rossmanith et al., 2014, pp. 8–9)

Rossmanith et al. (2014, pp. 18–19) insist that these unitary shared structures are easy to learn for infants, as developmental psychology has demonstrated “the impressive early achievements of infant learners,” i.e., their capacity to organize into “packages” the stream of experience thanks to statistical regularities and the structuring provided by caregivers.^22^ Since the action arc is characterized by a shared goal entailing interrelated actions of self and other—e.g., reading a book together from cover to cover—“sharing of affect goes hand in hand with, and is inseparable from, learning about the structure of the [overarching] action.” Infant teasing (Reddy, 2008; Reddy et al., 2013) can then be seen as the natural experiment through which infants document their possession of shared action schemas of this kind: “Once established as interpersonal routines, action structures lend themselves to be played with, e.g., introducing temporal variations that violate expectations (as in teasing)” (Rossmanith et al., 2014, p. 19).

In light of the idea that infants experience shared states as structured wholes of embodied states in self and other, it is not difficult to understand how the straightforward view accounts for better-known developmental findings on shared intentionality (Rakoczy, 2018; Tomasello, 2019). Infants have a “basic understanding of the basic structure of complementary roles” if they understand the common goal to which the different roles contribute (Rakoczy, 2018, p. 411). In reporting on the study by Warneken et al. (2006), Rakoczy (2018, p. 411) notes that 18-month-olds “respond in sophisticated ways when a partner fails in her fulfillment of the role: they try to reassign the role to her communicatively (by pointing out to her the object to be acted upon or the location where to act), help her to fulfill it and generally try to re-engage her *for the cooperation*” (my emphasis). Tomasello (2019, p. 197) interprets this finding by suggesting that “children, but not the chimpanzees, had created with their partner a joint agent ‘we’ whose breakdown they sought to repair” (*cf.* Vincini and Staiti, 2023).

Another example confirming that shared intentionality has to do with the overarching states identified by the straightforward approach consists in developmental studies on the division of resources. Tomasello (2019, p. 230) reviews findings that indicate that, whereas children rarely share toys they already individually possess, when “pairs of eighteen- and twenty-four-month-olds enter a room together and encounter a bowl of small, attractive toys (a situation somewhat reminiscent of chimpanzee foraging) […] they almost always divided up the toys in a relatively peaceful manner.” In other words, if the toys are something “we have found together,” then they belong to the context of the communal activity to which infants are sensitive. The idea that these global schemas for cooperation function in the child’s mind is further corroborated by findings such as that “three-year-olds are more likely to divide resources to especially benefit friends, people who have shared with them previously, and people who have shared with others previously” (Tomasello, 2019, pp. 230–231).

The advantage of the straightforward view over current psychological theories based on the assumption of multi-level recursive mindreading is easy to see. Both philosophers and developmentalists have argued that the existence of shared emotions, shared goals, and attention from as early as 3 months of age falsifies theories based on this assumption because the idea that infants so young may engage in multi-level recursive mindreading is untenable (Fantasia et al., 2014a; Rakoczy, 2018; Satne and Salice, 2020; León, 2021). This strong argument is corroborated by the reply of a developmental theorist like Rakoczy (2018, pp. 408, 415) who—in order to maintain the recursive mindreading assumption in some respects—seems obliged to affirm that there is no shared intentionality at 3 months because infants this young have no “grasp of other agents’ intentionality.” This reply does not seem to be convincing, because (i) there is ample evidence that infants at three months can perceive the goal-directedness of other agents (Vincini and Fantasia, 2022), and (ii) developmental psychologists do believe in the existence of sharing at this age (Zahavi and Rochat, 2015).

Nonetheless, although I am inclined to endorse the strong argument about falsification, since the goal of our examination is only to advocate the naturalistic character of the straightforward view, in this paper I propose a softer “parsimony argument.” It is possible to postulate that multi-level recursive mindreading at 3 months is underpinned by a specialized module that evolved in our ancestors in addition to the domain-general cognitive processes for the individuation of individual mental states described in section 3.2. This postulate is clearly less parsimonious than the straightforward view because, as section 4.2 explains, this view assumes that those domain-general processes suffice to generate shared states in social interaction. This parsimony argument is particularly appropriate for exhibiting the naturalistic character of the straightforward view, since it shows that the view solely relies on simple processes that are accepted by standard naturalistic theories.

The same dialectic can be pursued in relation to the postulate of a special, conceptually non-analyzable “sharing” phenomenal quality or “we-ness.” The straightforward view seems to be more accurate from the viewpoint of the experiential facts because, from an early age, humans experience shared emotions, shared goals, shared attention, etc., but, as Tollefsen (2015, pp. 33–34) has argued, nobody has ever experienced a special “sharing” or “we-ness” quality, which is indeed “mysterious.” In section 3, I suggested that the postulate of this seemingly mysterious quality derives from Cartesian eliminativism as soon as one tries to explain how mental states that can be had by only one individual are nonetheless experienced as shared. Here, I do not pursue the stronger “accuracy argument” concerning experiential facts, but I maintain the paper’s focus on parsimony. The postulate of a special phenomenal quality obliges us to imagine selective processes through which this quality evolved in our ancestors—processes whose details seem destined to remain unknown to a considerable extent. In contrast, the straightforward view solely relies on domain-general processes presupposed by most if not all naturalistic theories.

## 6. The functions of shared mental states

At this point, it seems essential to address an objection. Siposova and Carpenter (2019) devote an entire section of their paper to the functions of shared mental states because the fundamental methods and concepts of cognitive science require that mental states have functions. Otherwise, there would be no behavioral effects that could be measured (Sebanz and Knoblich, 2021). The objection is that the straightforward view may not be able to account for the functions of shared mental states. In order to neutralize this objection, we should start by considering the function of an individual mental state such as having an individual goal or intention. Then, in later steps, our consideration of the function of shared mental states will strengthen this paper’s parsimony argument and underline its importance.

If an intention is something that can be of any interest to cognitivist scientists—as it is—then, whatever brings about the intention, it must be something that brings about an action. As Searle (1980) explains, an individual raises her hand because she has the intention to raise it. Normally, *if there are no physical or psychological impediments*, then the fact that the individual *wants* to raise her hand is *sufficient* to cause the raising of her hand. Now, imagine that Max and Alex are two individuals of the species *Homo heidelbergensis* famously described by Tomasello (2016): they regularly carry out the joint action of hunting antelopes together, since this is the only way they can catch them. An uncontroversial function of a shared intention is that *a shared intention brings about a joint action*—which is usually expressed by saying that a shared intention enables coordination (Pacherie, 2013). When there are no physical or psychological impediments, a shared intention is *sufficient* to make Max and Alex perform the joint action. They decide to go hunting and they go.

In order to show how the straightforward view can account for this uncontroversial function of a shared intention, we should draw a contrast with Searle’s (1990) view of shared intentionality, since his view has a *prima facie* problem in accounting for this function. Famously, Searle (1990) assumes that a shared intention exists only in an individual brain, and thus that it can exist even if the individual brain is radically mistaken about the world—like a brain in a vat. Searle’s assumption has a notable consequence. On his assumption, one day Max can have—in his brain—the shared intention of hunting together with Alex, but *no joint action takes place* simply because Alex has no intention to go hunting—even though on that day there is no physical or psychological impediment and Max and Alex could very well go hunting if they wanted. Searle’s assumption implies that the shared intention exists even if Max’s beliefs about Alex are seriously mistaken, but this entails that a shared intention conceived *à la* Searle cannot fulfill the ordinary function of a shared intention in the absence of impediments, i.e., the function of bringing about a joint action.

The same problem applies to a broadly “Searlean” view according to which a shared emotion exists only in the mind, brain, or body of a single individual. Imagine an individual who mistakenly takes himself to be part of a shared emotion with his old friends at a reunion. On the Searlean view, the individual is indeed having a shared emotion and the nature of his shared emotion is not different from the one he would have if he were not mistaken and his friends had a corresponding shared emotion of the same kind. According to the Searlean view, the mistaken individual’s shared emotion exists because he feels that they are all having a great night, although he does not realize, e.g., that everyone else is bored and annoyed by how much he talks. The problem is that, if sometime later he tries to re-engage his old friends for a new reunion, he may find out—to his surprise—that nobody else is up for it. Indeed, one of the uncontroversial functions of shared emotions is group bonding (e.g., the group tends to meet again). As in the case of a shared intention, a Searlean view seems uncapable of accounting for this uncontroversial function of a shared mental state because it conceives of the shared mental state as existing within the boundaries of a single individual. In contrast, the straightforward view accounts well for this kind of function precisely because it conceives of the shared mental state as an event that is distributed among different individuals, and which is responsible for activities involving a plurality of individuals—a joint hunt or a new reunion (Krueger and Szanto, 2016).

However, in order to strengthen this paper’s parsimony argument, a further step is needed. Siposova and Carpenter (2019, pp. 262–263) propose a sophisticated Cartesian eliminativism that combines the assumption that a mental state can be had by only one individual with an idea that is advocated by supporters of the straightforward approach, such as Campbell (2005) and Eilan (n.d.). This is the idea that when individual A participates in a shared experience with individual B, what B experiences becomes a “constituent part” of A’s experience. This form of Cartesian eliminativism is an improvement over the Searlean approach because it can account for the uncontroversial functions of shared mental states. According to Siposova and Carpenter’s model, each individual who participates in a joint action has a shared intention that is exclusively her own, and each of these shared intentions is sufficient to cause the joint action. For example, there is a shared intention that only Max can have, but because this shared intention includes some elements in Alex’s mind as well, it is sufficient to cause the joint action; furthermore, there is a shared intention that only Alex can have, but because it includes some elements in Max’s mind too, it is also sufficient to cause the joint action. Despite its sophistication—or precisely because of it—Siposova and Carpenter’s model has a problem of redundancy: it postulates two numerically distinct shared intentions, both of which are sufficient causes of the same action.

In contrast, the straightforward view is a *non-redundant* explanation. It assumes that that there is nothing more than one shared state to fulfill the uncontroversial function of intention sharing. The straightforward analysis of sharing is that there is one overarching mental event that is owned by more than one individual. Therefore, the straightforward view is more parsimonious than Siposova and Carpenter’s view, which has to multiply the sufficient causes of a joint action.^23^

As a final step, I would like to underline the importance of this paper’s parsimony argument by considering the function of sharing in social cognition.^24^ In this other functional context, the *prima facie* problem of a theory based on multi-level recursive mindreading is circularity. A theory of this kind assumes that a shared state is grounded in reciprocal acts of recursive mindreading, but these are socio-cognitive acts directed at the other participant’s relevant mental states. Therefore, there seems to be little, if anything, that the shared mental state can add to social cognition that is not already provided by the socio-cognitive acts that ground the shared mental state.

The straightforward view approaches the function of sharing in social cognition in a radically different way (Fuchs and De Jaegher, 2009; Campbell, 2011; Satne, 2021; Vincini, 2021). As Campbell (2011) puts it, when an individual is in a joint attentional state with another person, she knows the other’s state of attention by means of “introspection.” Indeed, if the other’s state is one and the same as the experiential state I have, and I know about the state I have through introspection, then I also know the other’s state through introspection. The discussion of the cognitive-psychological processes underpinning the pre-reflective pre-delineation of a unitary shared mental state (section 4.2) allows us to see how the straightforward view avoids the circularity problem that derives from the assumption of multi-level recursive mindreading. Pre-reflective pre-delineation is the activation of a global pattern on the basis of some individual and social stimuli, e.g., the experience of some states of the self and the perception of some states of the other. However, the global pattern ordinarily includes many more states of the other than those that contributed to activating it, and this is why the pre-reflective pre-delineation of the shared mental state provides a substantial surplus of social cognition.^25^

As discussed in section 4.2, the straightforward view suggests that an exchange of attuned “communicative bids” can engender a “dyadic state of consciousness” between infant and caregiver. This attunement constitutes a similarity, or overlap, between the embodied states of self and other that tends to associate them together as a unitary whole. Moreover, the communicative bids of self and other are components of temporally extended shared patterns acquired through previous experience. Given the reciprocal familiarity between infant and primary caregiver, a mutual look can be sufficient to bring the content “we want to have fun together” out in the open for both participants. This simplicity of the straightforward account is the core of this paper’s argument. Our last step underlines the significance of this argument, since it shows that the less parsimonious assumption of multi-level recursive mindreading makes it difficult to account for sharing’s distinctive contribution to social cognition.

## 7. Conclusion

In the introduction to this paper, I referred to a variety of theoretical approaches that support the straightforward view, as well as to empirical studies that corroborate it in the fields of neuroscience, sociology, and social and developmental psychology. Unfortunately, this theoretical and empirical work is often dismissed or neglected due to an *a priori* attitude. This attitude consists in treating the Cartesian-eliminativist assumption that a mental state can be had by only one individual as a foundational assumption of psychology and cognitive science in general. What contradicts Cartesian eliminativism is excluded from the domain of natural science. The goal of this paper was to reveal the groundlessness of this *a priori* attitude.

In section 2, I indicated how Cartesian eliminativism generates (i) the postulate of a special ineffable quality when one pursues a systematic differentiation between shared intentionality and other social phenomena, and (ii) the postulate of multi-level recursive mindreading when one seeks to explain how the relevant functional features of other people’s mental states can be out in the open for each participant. In sections 3, 4, I showed that the straightforward view drops both of these postulates. In contrast to (i), the straightforward view describes the experience of shared intentionality as the experience of overarching mental states that are had by more than one individual. In contrast to (ii), the straightforward view suggests that low-level domain-general processes can suffice to bring a shared overarching state out in the open.

The straightforward view is a non-eliminativist, reductionist view according to which a shared mental state or experience is nothing other than the whole of the components distributed among the participants. The straightforward view is more parsimonious than influential naturalistic theories because it does not have to posit anything more than the low-level domain-general processes that these theories presuppose. The groundlessness of taking Cartesian eliminativism as a foundational assumption of cognitive science has been pursued by outlining the distinctive developmental predictions of the straightforward view (section 5) and by discussing how it can account for the functions of shared mental states (section 6).

What could be the impact on future research of showing the naturalistic viability of the straightforward view? First, since the straightforward view implies a hypothesis concerning the fundamental cognitive-psychological processes that associate the experience of self with the experience of others, the empirical corroboration of the straightforward view should also be pursued *indirectly* by testing strictly related theories that posit the functioning of the same cognitive-psychological processes in socio-cognitive phenomena—such as imitation development—which are intrinsically connected with (the development of) shared intentionality (Vincini and Gallagher, 2021). Second, the present contribution should promote the interdisciplinary study of the individuation of mental states and experiences—at both the pre-reflective and the reflective level, and in both the individual and the shared case—which could involve a variety of disciplines ranging from philosophy to sociology, and from neuroscience to anthropology (Barrett, 2017).

Finally, I hope that the naturalistic viability of the straightforward view of shared intentionality may inspire future empirical research by developmental psychologists with different theoretical inclinations. Those who already opposed the individualistic and intellectualist strictures of the Cartesian paradigm may find in the straightforward view a solid and parsimonious conceptual framework, which can nonetheless be developed in innovative ways. Those who have worked within the Cartesian paradigm—thus positing special phenomenal qualities and/or multi-level recursive mindreading—have also fruitfully employed different strands of the philosophical literature. I believe that this open-minded attention to philosophy manifests a serious attempt at finding a conceptual framework that may truly satisfy their insightful psychological intuitions. Does a thoroughly naturalistic version of straightforward realism render these intuitions better than Cartesianism?

## Data availability statement

The original contributions presented in the study are included in the article/Supplementary material, further inquiries can be directed to the corresponding author.

## Author contributions

The author confirms being the sole contributor of this work and has approved it for publication. However, the contributions of John Campbell and Naomi Elian to the creation of Figure 2 have been acknowledged in footnote 13.

## Funding

This research project has been made possible by the generous support of the Alexander von Humboldt Foundation.

## Conflict of interest

The author declares that the research was conducted in the absence of any commercial or financial relationships that could be construed as a potential conflict of interest.

## Publisher’s note

All claims expressed in this article are solely those of the authors and do not necessarily represent those of their affiliated organizations, or those of the publisher, the editors and the reviewers. Any product that may be evaluated in this article, or claim that may be made by its manufacturer, is not guaranteed or endorsed by the publisher.

## Footnotes

^1^See Carr (1986a,b), Salice and Schmid (2016), and Vincini and Staiti (2023) for an extensive discussion of exegetical questions concerning classical phenomenology.

^2^Cognitive scientists like Barrett (2017), Siposova and Carpenter (2019), and Tronick et al. (1998) talk about "mental states." I adopt this expression in order to make clear that this paper is about the kinds of phenomena that cognitive scientists designate with this expression. Following Barrett (2017, pp. 86, 103), I use the expression "mental event" to denote a mental phenomenon that "develops over time." In accordance with previous literature on the straightforward view and the 4E Cognition paradigm (Hutchins, 2014; Krueger, 2016; Gallagher, 2020), I use the term "process" to indicate a series of occurrences that are distributed over time and that are causally connected. Therefore, the term "process" also applies to phenomena such as Hebbian learning or association by contiguity or similarity. Finally, in line with certain philosophical literature (Husserl, Carr, Campbell, etc.), I also employ the term "experience." In this paper, goals, emotions, intentions, and episodes of attention are called "mental states," "mental events," "mental processes," and "experiences," choosing the expression that is deemed most appropriate in each context.

^3^In the discussion following her keynote address at the Collective Intentionality Conference 2020, Carpenter correctly indicated that many modern Western psychologists would consider this assumption to be a foundation of their theorizing.

^4^At the level of sharing, mutual knowledge would produce a qualitative coloration that is not present at the level of other social phenomena created by the interaction between individuals: "their perspectives and attention to the object of attention are colored by their mutual awareness of each other’s attention, and the experience differs qualitatively […] Their experience is qualitatively different from the individual experiences in the previous levels; […] it is colored by their direct mutual awareness of each other’s attention" (Siposova and Carpenter, 2019, p. 263).

^5^See also Eilan (n.d.).

^6^The complexity of this cognitive requirement is effectively characterized in the following passage from Siposova and Carpenter (2019, p. 264), which I quote only partially: "The levels have a cumulative structure in terms of the layers of knowledge involved. […] In the common, mutual, and shared levels, additionally, I know that each of us knows… […] In the mutual and shared levels, additionally, I know that we are both experiencing… […] In the shared level, additionally, we both know…."

^7^This example is inserted in this section because it is used by Barrett (2017) to illustrate an individual joy, although Barrett’s own description already points to how this emotion could easily become a shared pattern.

^8^Hoemann et al. (2019) suggest that that emotion words unify the distinct components of an emotion in virtue of "functional similarities." The pragmatic criteria of individuation are another point of convergence between otherwise very different approaches such as Barrett’s (2017), Scarantino’s (2014), and that of the classical phenomenologists (see Vincini, 2021).

^9^Phenomenologists insist that processes of reflective-linguistic individuation are not arbitrary and are based on processes of pre-reflective pre-delineation (Carr, 1986a,b; Brough, 2011; Zahavi, 2011). This explains why—as stated in the introduction—ordinary language in Western and non-Western cultures often individuates shared mental states in compliance with the pre-reflective processes described in section 4.2.

^10^"Cognitive processes are believed to emerge from complex interactions among very large numbers of neurons" (Hutchins, 2014, p. 37). This implies that the strengthening of a connection between two neural resources is such that the activation of one merely *tends to* activate the other. There are always other connections that can function as inhibitors in each particular case.

^11^At least initially, one might assume that, in the case of experiences that succeed one another, it is only the previous experience that facilitates the subsequent experience, and not vice versa. Yet, on a closer analysis, one should also consider the effect of the subsequent experience on the retention, or working memory, of the previous experience. The sense of what has just past seems to play a crucial role in the pre-reflective pre-delineation of experiences (Vincini, 2021).

^12^*Cf.* footnote 2.

^13^In an informal conversation on the straightforward view held during the recent "Minimal Forms of Shared Intentionality" workshop at TU Dortmund University (September 2022), John Campbell drew this graphic depiction of the view. On the right side of the picture, the line connecting the two individuals (A and B) was added by Naomi Eilan. The credit for this pictorial schema goes entirely to Campbell and Eilan, who drew and adjusted it during the same informal conversation.

^14^Obviously—except perhaps in some odd and unusual cases—the participants in a joint action do not share the *same* neural resources, but they do co-own one and the same overarching shared goal or intention, which includes the relevant neural resources in each participant as its components.

^15^Unfortunately, some commentators have overlooked the peculiar sense of reductionism from which Campbell (2005, 2011) differentiates the relational view of joint attention. A view can be reductionist in the sense relevant to this paper without being reductionist in the peculiar sense he specified. Furthermore, after one has clarified the distinction between reductionism and eliminativism, it becomes clear that Schmid’s version of the straightforward view is compatible with reductionism in the sense specified in this paragraph (the sense that matters for a naturalistic explanation). For a discussion of different forms of naturalism see Zahavi (2017).

^16^On how straightforward realism converges with some core ideas of Bratman’s account, see Gallagher (2020, 113) and Tollefsen (2015, 39–40); for its partial convergence with Gilbert and Tuomela, see Satne (2021, 517).

^17^For an interesting example of how the same kind of input—a feeling of "pleasant aggression"—can become, in different circumstances, part of individual and communal mental states, see Barrett (2017, pp. 141–142).

^18^"Infants experience their own behavior as a response to the emotional reactions that the other’s behavior has provoked in themselves; they also experience the other’s behavior as a response to their own active calls and solicitations" (Vincini and Fantasia, 2022, pp. 110–111).

^19^Not by chance, playful exchanges can be described both as emotion sharing and as joint activities (Eilan, n.d., 2020).

^20^As Crone (2021, p. 11819) puts it, "from a pragmatist perspective jointly shared meaning entails shared communicative aims."

^21^In the terminology of predictive coding, "individuals’ prior beliefs become more similar across couplings" (Vasil et al., 2020, p. 13). The "similar priors" that infant and caregiver quickly acquire constitute the distinct individual perspective on a shared experience.

^22^"One thing you are born with is a fundamental ability to learn from regularities and probabilities around you (In fact, you learn statistically even *in utero*)" (Barrett, 2017, p. 95).

^23^The same kind of parsimony advantage over Siposova and Carpenter’s model can be articulated with respect to the uncontroversial functions of emotion and attention sharing.

^24^See León (2021) for extensive references on the function of sharing in social cognition. For an account of the function of sharing in social cognition that can be shown to be substantially compatible with the straightforward view once the latter is clarified, see Seemann (2011).

^25^In this manner, the straightforward view also explains the fallibility of sharing as a mode of social cognition (Campbell, 2011). For example, when sharing a project with another person, we often assume that the other person is willing to pursue the project in a range of conditions that seem to be entirely unproblematic for us. It is only when the other person abandons the project due to some of these conditions arising that we must sadly realize that the range of unproblematic conditions was narrower for the other person or, in any case, different from our own.

## References

[ref1] Barrett L. F. (2017). How emotions are made: The secret life of the brain. New York, NY: Houghton-Mifflin-Harcourt.

[ref2] Bratman M. E. (1993). Shared intention. Ethics 104, 97–113. doi: 10.1086/293577

[ref3] Brough J. B. (2011). The most difficult of all phenomenological problems. Husserl Stud. 27, 27–40. doi: 10.1007/s10743-010-9082-6

[ref4] Butterfill S. Sinigaglia C. (2022). Towards a mechanistically neutral account of acting jointly: the notion of a collective goal. Mind 132, 1–29. doi: 10.1093/mind/fzab096

[ref5] Campbell J. (2002). Reference and consciousness. Oxford: Oxford University Press

[ref6] Campbell J. (2005). “Joint attention and common knowledge” in Joint attention: Communication and other minds. eds. Eilan N. Hoerl C. McCormack T. Roessler J. (New York: Oxford University Press), 287–297.

[ref7] Campbell J. (2011). “An object-dependent perspective on joint attention” in Joint attention: New developments in psychology, philosophy of mind, and social neuroscience. ed. Seemann A. (Cambridge, MA: MIT Press), 415–430.

[ref8] Carpenter M. Liebal K. (2011). “Joint attention, communication, and knowing together in infancy” in Joint attention: New developments in psychology, philosophy of mind, and social neuroscience. ed. Seemann S. (Cambridge, MA: MIT Press), 159–181.

[ref9] Carr D. (1986a). Cogitamus ergo sumus: the intentionality of the first-person plural. Monist 69, 521–533. doi: 10.5840/monist198669431

[ref10] Carr D. (1986b). Time, narrative, and history. Bloomington: Indiana University Press.

[ref11] Catmur C. Walsh V. Heyes C. (2009). Associative sequence learning: the role of experience in the development of imitation and the mirror system. Philos. Trans. R. Soc. Lond. Ser. B Biol. Sci. 364, 2369–2380. doi: 10.1098/rstb.2009.0048, PMID: 19620108 PMC2865072

[ref12] Cialdini R. B. Brown S. L. Lewis B. P. Luce C. Neuberg S. L. (1997). Reinterpreting the empathy–altruism relationship: when one into one equals oneness. J. Pers. Soc. Psychol. 73, 481–494. doi: 10.1037/0022-3514.73.3.481, PMID: 9294898

[ref13] Collins R. (2004). Interaction ritual chains. Princeton, NJ: Princeton University Press

[ref14] Corbetta D. (2021). Perception, action, and intrinsic motivation in infants’ motor-skill development. Curr. Dir. Psychol. Sci. 30, 418–424. doi: 10.1177/09637214211031939

[ref15] Cornejo C. (2008). Intersubjectivity as co-phenomenology: from the holism of meaning to the being-in-the-world-with-others. Integr. Psych. Behav. 42, 171–178. doi: 10.1007/s12124-007-9043-6, PMID: 18196357

[ref16] Crone K. (2021). Foundations of a we-perspective. Synthese 198, 11815–11832. doi: 10.1007/s11229-020-02834-6

[ref17] De Jaegher H. Di Paolo E. A. (2008). “Making sense in participation: an enactive approach to social cognition” in Enacting intersubjectivity: A cognitive and social perspective on the study of interactions. eds. Morganti F. Carassa A. Riva G. (Amsterdam: IOS Press), 33–47.

[ref18] Delafield-Butt J. T. Trevarthen C. (2015). The ontogenesis of narrative: from moving to meaning. Front. Psychol. 6:1157. doi: 10.3389/fpsyg.2015.01157, PMID: 26388789 PMC4557105

[ref19] Eilan N. (2007). “Consciousness, self-consciousness, and communication” in Reading Merleau-Ponty. On the phenomenology of perception. ed. Baldwin T. (New York: Routledge), 118–138.

[ref20] Eilan N. (2020). Other I’s, communication, and the second person. Inquiry, 1–23. doi: 10.1080/0020174X.2020.1788987

[ref21] Eilan N. (n.d.). Join Attention and the Second Person. Available at: https://warwick.ac.uk/fac/soc/philosophy/people/eilan/jaspup.pdf ().

[ref22] Fagard J. Esseily R. Jacquey L. O'Regan K. Somogyi E. (2018). Fetal origin of sensorimotor behavior. Front. Neurorobot. 12:23. doi: 10.3389/fnbot.2018.00023, PMID: 29875649 PMC5974044

[ref23] Fantasia V. De Jaegher H. Fasulo A. (2014a). We can work it out: an enactive look at 714 cooperation. Front. Psychol. 5. doi: 10.3389/fpsyg.2014.00874, PMID: 25152745 PMC4126490

[ref24] Fantasia V. Fasulo A. Costall A. López B. (2014b). Changing the game: exploring infants' participation in early play routines. Front. Psychol. 5:522. doi: 10.3389/fpsyg.2014.00522, PMID: 24936192 PMC4047965

[ref25] Fiebich A. (Ed.). (2020). Minimal cooperation and shared agency. Springer Series the Philosophy of Sociality. New York Springer.

[ref26] Fuchs T. De Jaegher H. (2009). Enactive intersubjectivity: participatory sensemaking and mutual incorporation. Phenomenol. Cogn. Sci. 8, 465–486. doi: 10.1007/s11097-009-9136-4

[ref27] Gallagher S. (2020). Action and interaction. Oxford: Oxford University Press

[ref28] Gallagher S. Zahavi D. (2020). The phenomenological mind, 3rd. London: Routledge.

[ref29] Gallese V. Caruana F. (2016). Embodied simulation. Beyond the expression/experience dualism of emotions. Trends Cogn. Sci. 20, 397–398. doi: 10.1016/j.tics.2016.03.010, PMID: 27101879

[ref30] Gatyas M. (2022). Emotion sharing as empathic. Philos. Psychol. 36, 85–108. doi: 10.1080/09515089.2022.2038121

[ref31] Gonzalez-Castillo J. Saadb Z. S. Handwerkera D. A. Inatic S. J. Brenowitza N. Bandettini P. A. (2012). Tracking ongoing cognition in individuals using brief, whole-brain functional connectivity patterns. Proc. Natl. Acad. Sci. 112, 8762–8767. doi: 10.1073/pnas.1501242112, PMID: 26124112 PMC4507216

[ref32] Hall G. (1994). “Pavlovian conditioning: laws of association” in Animal learning and cognition. ed. Mackintosh N. J. (San Diego: Academic Press), 15–43.

[ref33] Harré R. (Ed.) (1986). The social construction of emotions. Oxford, UK: Blackwell.

[ref34] Heyes C. M. (1994). Reflections on self-recognition in primates. Anim. Behav. 47, 909–919. doi: 10.1006/anbe.1994.1123

[ref35] Heyes C. M. Ray E. D. (2000). “What is the significance of imitation in animals?” in Advances in the study of behavior. eds. Slater P. J. B. Rosenblatt J. S. Snowdon C. T. Roper T. J., vol. 29 (San Diego, CA: Academic Press), 215–245.

[ref36] Hobson R. P. Hobson J. (2011). “Joint attention or joint engagement? Insights from autism” in Joint attention: New developments in psychology, philosophy of mind, and social neuroscience. ed. Seemann A. (Cambridge, MA: MIT Press), 115–136.

[ref37] Hoemann K. Xu F. Barrett L. F. (2019). Emotion words, emotion concepts, and emotional development in children: a constructionist hypothesis. Dev. Psychol. 55, 1830–1849. doi: 10.1037/dev0000686, PMID: 31464489 PMC6716622

[ref38] Husserl E. (1962). Phänomenologische Psychologie. The Hague: Nijhoff.

[ref39] Husserl E. (1973a). Zur Phänomenologie der Intersubjektivität II. The Hague: Nijhoff

[ref40] Husserl E. (1973b). Zur Phänomenologie der Intersubjektivität III. The Hague: Nijhoff.

[ref001] Husserl E. (1999). Cartesian Meditations: An Introduction to Phenomenology. The Hague: Martinus Nijhoff.

[ref41] Hutchins E. (2014). The cultural ecosystem of human cognition. Philos. Psychol. 27, 34–49. doi: 10.1080/09515089.2013.830548

[ref42] Jankovic M. Ludwig K. (Eds.). (2018). The Routledge handbook of collective intentionality. London: Routledge.

[ref43] Krueger J. (2016). “The affective ‘we’. Self-regulation and shared emotions” in The phenomenology of sociality: discovering the ‘we’. eds. Szanto T. Moran D. (London/New York: Routledge), 263–276.

[ref44] Krueger J. Szanto T. (2016). Extended emotions. Philos. Compass 11, 863–878. doi: 10.1111/phc3.12390

[ref45] León F. (2021). Joint attention without recursive mindreading: on the role of second-person engagement. Philos. Psychol. 34, 550–580. doi: 10.1080/09515089.2021.1917533

[ref46] Li W. Woudstra M. J. Branger M. C. E. Wang L. Alink L. R. A. Mesman J.. (2019). The effect of the still-face paradigm on infant behavior: a cross-cultural comparison between mothers and fathers. Infancy 24, 893–910. doi: 10.1111/infa.12313, PMID: 32677359

[ref47] Liszkowski U. Carpenter M. Henning A. Striano T. Tomasello M. (2004). Twelve-month-olds point to share attention and interest. Dev. Sci. 7, 297–307. doi: 10.1111/j.14677687.2004.00349.x, PMID: 15595371

[ref48] Lloyd D. (2017). Protention and predictive processing: the wave of the future. Constr. Found. 13, 98–99.

[ref49] Lövdén M. Garzón B. Lindenberger U. (2020). Human skill learning: expansion, exploration, selection, and refinement. Curr. Opin. Behav. Sci. 36, 163–168. doi: 10.1016/j.cobeha.2020.11.002

[ref51] Martens J. H. (2021). Habit and skill in the domain of joint action. Topoi 40, 663–675. doi: 10.1007/s11245-020-09732-z

[ref52] Mesman J. Van IJzendoorn M. H. Bakermans-Kranenburg M. J. (2009). The many faces of the still-face paradigm: a review and meta-analysis. Dev. Rev. 29, 120–162. doi: 10.1016/j.dr.2009.02.001

[ref53] Newen A. Welpinghus A. Juckel G. (2015). Emotion recognition as pattern recognition: the relevance of perception. Mind Lang. 30, 187–208. doi: 10.1111/mila.12077

[ref54] Northoff G. (2016). Slow cortical potentials and inner time consciousness—a neuro-phenomenal hypothesis about the width of present. Int. J. Psychophysiol. 103, 174–184. doi: 10.1016/j.ijpsycho.2015.02.012, PMID: 25678022

[ref55] Pacherie E. (2013). Intentional joint agency: shared intention lite. Synthese 190, 1817–1839. doi: 10.1007/s11229-013-0263-7

[ref56] Papousek M. (2007). Communication in early infancy: an arena of intersubjective learning. Infant Behav. Dev. 30, 258–266. doi: 10.1016/j.infbeh.2007.02.003, PMID: 17363062

[ref57] Prochazkova E. Kret M. E. (2017). Connecting minds and sharing emotions through mimicry: a neurocognitive model of emotional contagion. Neurosci. Biobehav. Rev. 80, 99–114. doi: 10.1016/j.neubiorev.2017.05.013, PMID: 28506927

[ref58] Rakoczy H. (2018). “Development of collective intentionality” in The Routledge handbook of collective intentionality. eds. Jankovic M. Ludwig K. (London: Routledge), 407–419.

[ref59] Reddy V. (2008). How infants know minds. Cambridge, MA: Harvard University Press.

[ref60] Reddy V. Liebal K. Hicks K. Jonnalagadda S. Chintalapuri B. (2013). The emergent practice of infant compliance: an exploration in two cultures. Dev. Psychol. 49, 1754–1762. doi: 10.1037/a0030979, PMID: 23231690

[ref61] Rimé B. (2007). The social sharing of emotion as an Interface between individual and collective processes in the construction of emotional climates. J. Soc. Issues 63, 307–322. doi: 10.1111/j.1540-4560.2007.00510.x

[ref62] Rizzolatti G. Sinigaglia C. (2016). The mirror mechanism: a basic principle of brain function. Nat. Rev. Neurosci. 17, 757–765. doi: 10.1038/nrn.2016.135, PMID: 27761004

[ref63] Rochat P. (2003). Five levels of self-awareness as they unfold early in life. Conscious. Cogn. 12, 717–731. doi: 10.1016/S1053-8100(03)00081-3, PMID: 14656513

[ref64] Rossmanith N. Costall A. Reichelt A. F. López B. Reddy V. (2014). Jointly structuring triadic spaces of meaning and action: book sharing from 3 months on. Front. Psychol. 5:1390. doi: 10.3389/fpsyg.2014.01390 25540629 PMC4261719

[ref65] Salice A. (2015). Sharing an emotion: a Schelerian approach. Thaumazein 3, 83–102. doi: 10.13136/thau.v3i0.39

[ref66] Salice A. Miyazono K. (2020). Being one of us. Group identification, joint actions, and collective intentionality. Philos. Psychol. 33, 42–63. doi: 10.1080/09515089.2019.1682132

[ref67] Salice Alessandro Schmid Hans Bernhard (Eds.) (2016). The phenomenological approach to social reality: History, concepts, problems. Dordrecht: Springer

[ref68] Salmela M. (2012). Shared emotions. Philos. Explor. 15, 33–46. doi: 10.1080/13869795.2012.647355

[ref69] Satne G. (2021). Understanding others by doing things together: an enactive account. Synthese 198, 507–528. doi: 10.1007/s11229-020-02692-2

[ref70] Satne G. Salice A. (2020). “Shared intentionality and the cooperative evolutionary hypothesis” in Minimal cooperation and shared agency. ed. Fiebich A. (Dordrecht: Springer), 71–92.

[ref71] Scarantino A. (2014). “The motivational theory of emotions” in Moral psychology and human agency: Philosophical essays on the science of ethics. eds. D'Arms J. Jacobson D. (New York, NY: Oxford University Press), 156–185.

[ref72] Scheler M. (1973). Formalism in ethics and non-formal ethics of values. Evanston Northwestern University Press.

[ref73] Scheler M. (2008). The nature of sympathy. London Transaction Publishers.

[ref74] Scheler M. (2009). The human place in the cosmos. Northwestern University Press: Evanston, IL.

[ref75] Schilbach L. Timmermans B. Reddy V. Costall A. Bente G. Schlicht T.. (2013). Toward a second-person neuroscience. Behav. Brain Sci. 36, 393–414. doi: 10.1017/S0140525X12000660, PMID: 23883742

[ref76] Schmid H. B. (2005). Wir-Intentionalität: Kritik des Ontologischen Individualismus und Rekonstruktion der Gemeinschaft. Freiburg, Germany: Karl Alber.

[ref77] Schmid H. B. (2009). Plural action. Essays in philosophy and social science. Dordrecht: Springer

[ref78] Schweikard D. P. Schmid H. B. (2021). Collective intentionality. The stanford encyclopedia of philosophy. Fall 2021 edition. Available at: https://plato.stanford.edu/archives/fall2021/entries/collective-intentionality/

[ref79] Searle J. R. (1980). The intentionality of intention and action. Cogn. Sci. 4, 47–70. doi: 10.1207/s15516709cog0401_3

[ref80] Searle J. (1990). “Collective intentions and actions” in Intentions in communication. eds. Cohen P. Morgan J. Pollack M. E. (Cambridge, MA: Bradford Books, MIT Press)

[ref81] Sebanz N. Knoblich G. (2021). Progress in joint-action research. Curr. Dir. Psychol. Sci. 30, 138–143. doi: 10.1177/0963721420984425

[ref82] Seemann A. (2011). “Joint attention: toward a relational account” in Joint attention: New developments in psychology, philosophy of mind, and social neuroscience. ed. Seemann A. (Cambridge MA: The MIT Press), 183–202.

[ref83] Sinigaglia C. Butterfill S. A. (2022). Motor representation in acting together. Synthese 200, 1–6. doi: 10.1007/s11229-022-03539-8

[ref84] Siposova B. Carpenter M. (2019). A new look at joint attention and common knowledge. Cognition 189, 260–274. doi: 10.1016/j.cognition.2019.03.019, PMID: 31015079

[ref85] Smith E. R. Seger C. R. Mackie D. M. (2007). Can emotions be truly group level? Evidence regarding four conceptual criteria. J. Pers. Soc. Psychol. 93, 431–446. doi: 10.1037/0022-3514.93.3.431, PMID: 17723058

[ref86] Stein E. (2000). Philosophy of psychology and the humanities (M. Sawicki, Ed., M. C. Baseheart and M. Sawicki, Trans.). Washington, DC: ICS Publication.

[ref87] Stern D. N. (1990). Diary of a baby. New York Basic Books.

[ref88] Stern D. N. Bruschweiler-Stern N. Harrison A. M. Lyons-Ruth K. Morgan A. C. Nahum J. P.. (1998). The process of therapeutic change involving implicit knowledge: some implications of developmental observations for adult psychotherapy. Infant Ment. Health J. 19, 300–308. doi: 10.1002/(SICI)1097-0355(199823)19:3<300::AID-IMHJ5>3.0.CO;2-P

[ref89] Theiner G. (2018). “Groups as distributed cognitive systems” in The Routledge handbook of collective intentionality. eds. Jankovic M. Ludwig K. (New York, NY: Routledge), 233–248.

[ref90] Tollefsen D. (2002). Collective intentionality and the social sciences. Philos. Soc. Sci. 32, 25–50. doi: 10.1177/004839310203200102

[ref91] Tollefsen D. (2015). Groups as agents. Cambridge, UK: Polity Press.

[ref92] Tomasello M. (2016). A natural history of human morality. Cambridge, MA: Harvard University Press

[ref93] Tomasello M. (2019). Becoming human: A theory of ontogeny. Cambridge, MA Harvard University Press.

[ref94] Tronick E. Z. Bruschweiler-Stern N. Harrison A. M. Lyons-Ruth K. Morgan A. C. Nahum J. P.. (1998). Dyadically expanded states of consciousness and the process of therapeutic change. Infant Ment. Health J. 19, 290–299. doi: 10.1002/(SICI)1097-0355(199823)19:3<290::AID-IMHJ4>3.0.CO;2-Q

[ref95] Varela F. J. (1999) The specious present: a neurophenomenology of time consciousness. In: Petitot J. Varela F. J. Pachoud B. Roy J.-M. Naturalizing phenomenology: Issues in contemporary phenomenology and cognitive science. Stanford University Press, Stanford: 266–329.

[ref96] Vasil J. Badcock P. B. Constant A. Friston K. Ramstead M. J. D. (2020). A world unto itself: human communication as active inference. Front. Psychol. 11:417. doi: 10.3389/fpsyg.2020.00417, PMID: 32269536 PMC7109408

[ref97] Vincini S. (2020). The pairing account of infant social perception. J. Conscious. Stud. 27, 173–205. Available at: https://www.ingentaconnect.com/content/imp/jcs/2020/00000027/f0020001/art00008

[ref98] Vincini S. (2021). Pairing and sharing: the birth of the sense of us. Phenomenol. Cogn. Sci. doi: 10.1007/s11097-021-09793-4

[ref99] Vincini S. (in press). “Can interactional approaches solve the empathy-sharing conundrum?” in The empathic understanding of persons, art, and literature. eds. Werner C. Petraschka T. (Oxfordshire: Routledge)

[ref100] Vincini S. Fantasia V. (2022). Rich or lean? A phenomenological alternative for explaining early social cognition. Riv. Internazionale Filos. Psicol. 3, 108–125. Available at: https://www.rifp.it/ojs/index.php/rifp/article/view/rifp.2022.0011/1223

[ref101] Vincini S. Gallagher S. (2021). Developmental phenomenology: examples from social cognition. Cont. Philos. Rev. 54, 183–199. doi: 10.1007/s11007-020-09510-z

[ref102] Vincini S. Jhang Y. (2018). Association but not recognition: an alternative model for differential imitation from 0 to 2 months. Rev. Philos. Psychol. 9, 395–427. doi: 10.1007/s13164-017-0373-0

[ref103] Vincini S. Jhang Y. Buder E. H. Gallagher S. (2017). Neonatal imitation: theory, experimental design, and significance for the field of social cognition. Front. Psychol. 8:1323. doi: 10.3389/fpsyg.2017.01323, PMID: 28824502 PMC5543082

[ref104] Vincini S. Staiti A. (2023). “Tomasello, Husserl, and the cognitive foundations of morality” in Ethics and empathy. eds. Ferrarello S. Englander M. (Lanham: Rowman & Littlefield), 207–231.

[ref105] Vogel E. H. Ponce F. P. Wagner A. R. (2019). The development and present status of the SOP model of associative learning. Q. J. Exp. Psychol. 72, 346–374. doi: 10.1177/1747021818777074, PMID: 29741452

[ref106] von Scheve C. Ismer S. (2013). Towards a theory of collective emotions. Emot. Rev. 5, 406–413. doi: 10.1177/1754073913484170

[ref107] Walsh P. J. (2020). Intercorporeity and the first-person plural in Merleau-Ponty. Cont. Philos. Rev. 53, 21–47. doi: 10.1007/s11007-019-09480-x

[ref108] Warneken F. Chen F. Tomasello M. (2006). Cooperative activities in young children and chimpanzees. Child Dev. 77, 640–663. doi: 10.1111/j.1467-8624.2006.00895.x, PMID: 16686793

[ref109] Zahavi D. (2011). Objects and levels: reflections on the relation between time-consciousness and self-consciousness. Husserl Stud. 27, 13–25. doi: 10.1007/s10743-010-9084-4

[ref110] Zahavi D. (2014). Self and other: Exploring subjectivity, empathy, and shame. Oxford, UK: Oxford University Press

[ref111] Zahavi D. (2017). Husserl’s legacy: Phenomenology, metaphysics, and transcendental philosophy. Oxford: Oxford University Press.

[ref112] Zahavi D. (2019). Second-person engagement, self-alienation, and group-identification. Topoi 38, 251–260. doi: 10.1007/s11245-016-9444-6

[ref113] Zahavi D. Rochat P. (2015). Empathy≠sharing: perspectives from phenomenology and developmental psychology. Conscious. Cogn. 36, 543–553. doi: 10.1016/j.concog.2015.05.008, PMID: 26070850

[ref114] Zickfeld J. H. Schubert T. W. Seibt B. Fiske A. P. (2017). Empathic concern is part of a more general communal emotion. Front. Psychol. 8:723. doi: 10.3389/fpsyg.2017.00723, PMID: 28539901 PMC5423947

